# Distribution Patterns Predict Individual Specialization in the Diet of Dolphin Gulls

**DOI:** 10.1371/journal.pone.0067714

**Published:** 2013-07-02

**Authors:** Juan F. Masello, Martin Wikelski, Christian C. Voigt, Petra Quillfeldt

**Affiliations:** 1 Max Planck Institute for Ornithology, Vogelwarte Radolfzell, Radolfzell, Germany; 2 Evolutionary Ecology research Group, Leibniz Institute for Zoo and Wildlife Research, Berlin, Germany; University of Plymouth, United Kingdom

## Abstract

Many animals show some degree of individual specialization in foraging strategies and diet. This has profound ecological and evolutionary implications. For example, populations containing diverse individual foraging strategies will respond in different ways to changes in the environment, thus affecting the capacity of the populations to adapt to environmental changes and to diversify. However, patterns of individual specialization have been examined in few species. Likewise it is usually unknown whether specialization is maintained over time, because examining the temporal scale at which specialization occurs can prove difficult in the field. In the present study, we analyzed individual specialization in foraging in Dolphin Gulls *Leucophaeus scoresbii*, a scavenger endemic to the southernmost coasts of South America. We used GPS position logging and stable isotope analyses (SIA) to investigate individual specialization in feeding strategies and their persistence over time. The analysis of GPS data indicated two major foraging strategies in Dolphin Gulls from New I. (Falkland Is./Islas Malvinas). Tagged individuals repeatedly attended either a site with mussel beds or seabird and seal colonies during 5 to 7 days of tracking. Females foraging at mussel beds were heavier than those foraging at seabird colonies. Nitrogen isotope ratios (δ^15^N) of Dolphin Gull blood cells clustered in two groups, showing that individuals were consistent in their preferred foraging strategies over a period of at least several weeks. The results of the SIA as well as the foraging patterns recorded revealed a high degree of specialization for particular feeding sites and diets by individual Dolphin Gulls. Individual differences in foraging behavior were not related to sex. Specialization in Dolphin Gulls may be favored by the advantages of learning and memorizing optimal feeding locations and behaviors. Specialized individuals may reduce search and handling time and thus, optimize their energy gain and/or minimize time spent foraging.

## Introduction

Many animals show some degree of individual specialization in diet. Specialized individuals occupy niches that are subsets of a more generalist population niche. In this way, co-occurring individuals actively select different prey from their shared environment [1]. The observed dietary differences within a population are often largely a result of sex- or age-related differences in size or experience (e.g. [2], [3]). However, individuals in many species also show differences in diet preference independent of age or sex (e.g. [4], [5]). Two recent reviews [1], [6] suggested that individual specialization is a widespread but under-appreciated phenomenon.

Individual specialization has important implications. The ecological niche of a species is usually described assuming conspecific individuals to be ecologically equivalent [6]. However, a population containing distinct individual foraging strategies presents a fundamentally different ecological context. For example, such a population will respond in more than one way to changes in the environment, have a lower degree of intraspecific competition, and individuals within the same population may be subject to diet-specific selective pressures. Thus, individual specialization may also affect the population’s capacity to withstand resource fluctuations [7], to diversify and presumably also to speciate.

Not only has individual specialization received attention in only a few species, but it is also often unknown whether specializations are maintained over time. To examine the temporal scale at which specialization occurs can prove difficult in the field. However, the measurement of naturally occurring stable isotopes in consumers and their prey has been found particularly useful in elucidating differences in diet and distribution among individuals (e.g. [8]–[13]). Studies using stable isotope analyses avoid many problems associated with conventional approaches in studying seabird diets, such as biases due to differential detectability of prey (e.g. [14]) and the need for obtaining large sample sizes with invasive sampling methods. Stable isotopes provide an integrated measure of assimilated dietary nutrients, with a time scale of hours to years depending on the isotopic turnover rate of the tissue sampled.

In the present study, we analyzed individual specialization of foraging behavior in Dolphin Gulls *Leucophaeus scoresbii*, a scavenger endemic to the southernmost coasts of South America. The diet described for this species mostly consists of food dropped by penguins, cormorants and petrels while these birds are feeding their chicks (for a review see [15]). Other described food items include intertidal mussels, crustaceans and polichaetes, invertebrates washed ashore after storms, Southern Sea Lions *Otaria flavescens* feces, rests of seabird eggs stolen by other avian predators from several penguin and cormorant species, insects, algae, carrion, and food derived from human activities ([15] and references therein, [16]–[20]). The feeding behavior influences the distribution of Dolphin Gulls, which is highly dependent on food from seabird colonies, e.g. Imperial Shags *Leucocarbo atriceps*, and Magellanic Penguins *Spheniscus magellanicus* (e.g. [17], [21]). At Punta Tombo, Argentina, individual birds were seen repeatedly at the same place, suggesting individuals have an affinity for particular foraging locations [15], [17].

To analyze individual specialization in Dolphin Gulls, we here used GPS position logging and stable isotope analyses of red blood cells to determine the diet assimilated over a period of several weeks prior to sampling. In particular, we ask whether:

1. Individuals are specialized in particular feeding sites and diets,
2. Sexual differences can explain patterns of specialization,
3. Specialization influences the body mass or breeding parameters of the individuals,
4. Specialization observed during several days (GPS loggers) is maintained over several weeks (stable isotope values).

## Methods

### Study Site and Study Species

**Figure 1:**
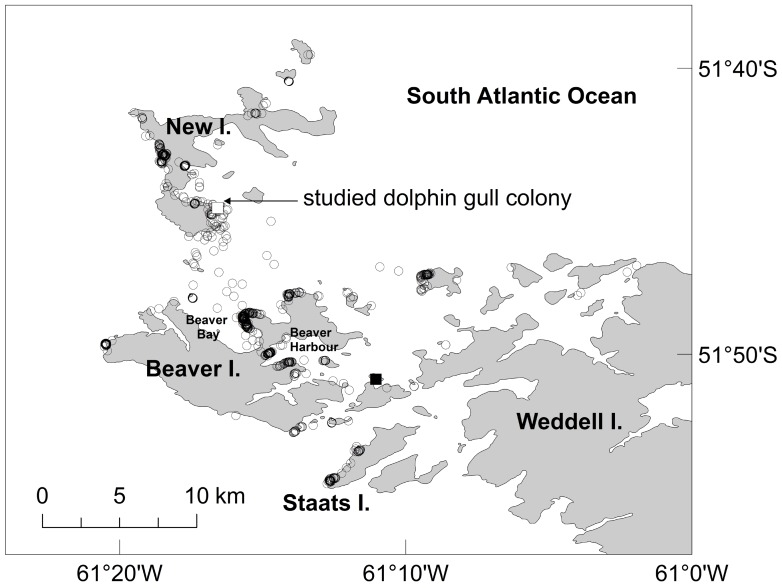
Global Positioning System (GPS) locations of Dolphin Gulls *Leucophaeus scoresbii.* The studied breeding colony at New Island (51°43′S, 61°18′W; 76 nests), Falkland Islands/Islas Malvinas, in the South-western Atlantic is indicated with a white square and an arrow. A second Dolphin Gull colony (20 nests) in the region is marked with a black square. Data correspond to eight incubating females and eight incubating males tagged during 2–9 January 2009. GPS fixes are marked with a circle.

Fieldwork was carried out at New Island Nature Reserve, Falkland Islands/Islas Malvinas, (51°43′S, 61°18′W, Fig. 1), southwestern Atlantic Ocean, between 2 and 9 January 2009. In December 2010, we surveyed from a sailing boat all the major feeding areas visited by the Dolphin Gull from New Island (File S1, Fig. S1). The identity, location and size of seabird and mammal colonies (i.e. potential food sources) are presented in the File S5, Table S1.

The Falkland/Malvinas Current generates an area of ocean water upwelling just west of New Island. This area of increased productivity (e.g. [22], [23]) attracts several seal and seabird species that breed in colonies distributed over New I. (2,011 ha, 84 km of coastline; [24], [25]). Rockhopper Penguins *Eudyptes chrysocome* breed in 5 colonies at New I., the two main ones having about 5,000 and 3,000 breeding pairs [File S1, Fig. S1; [24]−[26]. Magellanic Penguins are widespread as nesting birds in New I., with >3,700 nesting birds [24], [25]. Gentoo Penguins *Pygoscelis papua* breed in two areas on New I., one at the North End (>6,000 pairs), and one at the South End (>500 pairs; File S1, Fig. S1; [24]−[2426]). Imperial Shags breed on New I. at three sites, but the majority (>3,000 nest) is found in the west of the island (File S1, Fig. S1; [24], [25]). More than 14,000 pairs of Black-browed Albatross *Thalassarche melanophrys* breed at a mixed seabird colony on New I. (File S1, Fig. S1; [27]. Other seabirds breeding on New I. include Thin-billed Prions *Pachyptila belcheri* (>2 million pairs), White-chinned Petrels *Procellaria aequinoctialis* (40 pairs), and Giant Petrel *Macronectes giganteus* (30 to 40 pairs; [24], [26]). The total number of Fur Seals *Arctocephalus australis* at New I. has been estimated at around 2,000 animals [24]. Dense beds or mats of Blue Mussel *Mytilus edulis chilensis* are common on the coasts of the Falkland Islands/Islas Malvinas but largely confined to sheltered bays [16]. Very large Blue Mussel beds are located close to New I., particularly in two sheltered bays of Beaver I., namely Beaver Harbour and Beaver Bay (Fig. 1; File S1, Fig. S1). On New I., it is also possible to find Blue Mussel beds in several sheltered sectors of the east coast but they are much smaller than the ones found on Beaver Island (File S1, Fig. S1).

Dolphin Gulls nest in close proximity to each other in dense colonies. Breeding sites are associated with other colonial seabirds and marine mammals, mainly cormorants, penguins, gulls and sea lions. On the Atlantic coast of Argentina, 26 colonies with an estimate of fewer than 700 breeding pairs in total are found from Tierra del Fuego north to Punta Tombo [28]. The population size of the Falkland Islands/Islas Malvinas is unknown (but less than 500 breeding pairs from our own observations). In Chile, the species is found north to Chiloe Island [29]. Dolphin Gulls lay a clutch of one to three eggs, and timing of breeding coincides with the peak breeding activity of the associated colonial seabirds [17].

Aspects of the feeding ecology of Dolphin Gulls have been described at different locations throughout its range, although the information is largely anecdotal and descriptive. Dolphin Gull diet can include food dropped by other colonial seabirds, feces of marine mammals, intertidal mussels, algae, and invertebrates washed ashore after storms [15−21], . Movements of Dolphin Gulls have been mapped by radio tracking at Punta Tombo, where 99% of locations were within 2.4 km of the Dolphin Gull colony [15].

### Instrumentation and Fieldwork Procedures

Dolphin Gulls were caught at their breeding colony at New I. (Fig. 1), using an incubation trap (described in [37]; see also [15]) on 2 January 2009. During that breeding season, the colony consisted of 76 nests. GPS/acceleration loggers (e-obs GmbH, Munich, Germany) were deployed on 10 female and 10 male incubating Dolphin Gulls using Tesa® tape. We followed the long-term attachment method (Method 2) of recording devices to penguins and other seabirds developed by [38] (see also [25]), with devices placed to the center of the back. These loggers provide detailed position (longitude, latitude), as well as acceleration data and time of day. The loggers weighed 20 g and measured 45×23×20 mm, representing 3.6% of the adult body mass (mean 550±15.7 g, range 450 to 630 g). We took blood samples for molecular sexing and isotope analyses. Later on in the lab (Max Planck Institute for Ornithology, Seewiesen), all birds were sexed following standard molecular methods [39]. This was done to confirm an equal number of each sex in our sample. Body mass was recorded using a digital balance to the nearest 1 gram. Additionally, we recorded clutch size (the number of eggs laid per nest). Birds were released 10 metres from their nest, and all returned to their eggs within 2−3 minutes.

The sampling interval for the GPS data was set to 900 s to conserve battery and allow the data logger to work several days. To download the data, we walked to within 100−200 m of the colony several times a day, for 8 days in total, with a handheld receiver base station. The GPS/acceleration loggers were programmed to contact the base station at a frequency of 868.3 MHz every 30 seconds (for more details see [40]). Whenever a bird was nearby (as determined retrospectively from logged GPS data), we received and downloaded all its data. The data were subsequently uploaded to Movebank 〈http://www.movebank.org〉, a global repository of animal movement data.

Birds were recaptured in their nests and loggers recovered after 3–7 days of deployment. In two cases, the birds lost their data logger and no data could be downloaded. In one case, the logger was detached from the bird and lay in the nest during the entire study period. This logger was recovered but its data was of no use. In another case, the data logger malfunctioned and only partial data could be recovered.

### Analyses of Spatial and Temporal Data

Positional data obtained from GPS loggers (geographic coordinate system WGS1984) were used to plot and analyze, with the use of ArcGIS 9.3, the trips performed by the birds. Trip length was calculated as the total cumulative linear distance between all positional fixes along the foraging trip, outside of the colony. For each trip, the maximum distance from the colony was calculated as the linear grand circle distance between the furthest point of the plotted trip and the geographical coordinates of the departure colony, determined by GPS. Trip duration was determined as the time lapse between departure and return from the colony. As Dolphin Gulls are slightly sexual dimorphic [17], data were checked for sexual differences (Table 1), but no differences were detected. Therefore, we here pooled the data of males and females. Throughout this study means are given ± standard deviations (SD) unless stated otherwise. The significance level used is *P*<0.05.

**Table 1:** Parameters of foraging trips of Dolphin Gulls *Leucophaeus scoresbii*.

	*n*			Trip length	Maximum distance from colony	Trip duration (min)	N° of trips per day
Mussel feeders	6			21.6±3.6 (17−26.3)	9.6±1.4 (7.8−11.5)	476±238 (255−930)	1.5±0.6 (1−2)
Colony feeders	10			18.2±10.7 (3.2−41.4)	7.8±4.9 (1.3−18.7)	357±165 (90−638)	2±0.5 (1−3)
*t*-test for Equality of Means				t = 0.7	t = 0.9	t = 1.2	t = -1.8
				P = 0.47	P = 0.40	P = 0.25	P = 0.09
Males	8			17.4±7.1 (3.2−24.3)	7.8±3.4 (1.3−10.7)	332±152 (90−533)	1.9±0.7 (1−3)
Females	8			21.5±10.0 (8.4−41.3)	9.1±4.7 (4.1−18.7)	472±220 (240−930)	1.8±0.5 (1−2)
*t*-test for Equality of Means				t = 0.9	t = 0.7	t = 1.5	t = -0.4
				P = 0.37	P = 0.53	P = 0.16	P = 0.73

Parameters were determined using GPS loggers during the incubation period at New Island (51°43′S, 61°18′W), Falkland Islands/Islas Malvinas, in the southwestern Atlantic Ocean. Median data for each individual were compared, i.e. one data point per individual was used to determine means, standard deviations and ranges, and to test for differences between mussel and colony feeders. One-Sample Kolmogorov-Smirnov Test (all P>0.2) and visual inspection of histograms revealed that the parameters were normally distributed. Distances are given in kilometers.

### Kernel Distribution

The nonparametric fixed kernel density estimator was used to determine the 50, 60, 70, 80, 90 and 95% density contour areas (the estimated foraging range; [41]). Kernel densities indicate where, during a foraging trip, birds spent most of their time [41]. Density contours corresponding to kernels were calculated for locations in a Lambert Equal-Area Azimuthal projection centered on the South Pole, as in [42]. ArcGIS 9.3 was used for calculations together with Hawth's Analysis Tools [43]. GPS data-points at the colony were excluded from our analyses because dolphin gulls do not forage at their breeding colony on New I. or in areas very close to it.

### Stable Isotope Analysis

Stable isotope analysis (SIA) constitutes a powerful instrument for extending our knowledge about the foraging behavior of birds (e.g. [3], [12], [25], ). This technique takes advantage of the fact that the isotopes of various elements are distributed in the environment unevenly but according to specific rules. Thus, the ratios of the naturally occurring isotopes of nitrogen (^15^N/^14^N; δ^15^N) and carbon (^13^C/^12^C; δ^13^C) in food webs and along ecological gradients change in a predictable manner [50]. The carbon and nitrogen isotope composition (δ^13^C and δ^15^N) differs between organisms and their diets because of a selective retention of the heavy isotope and excretion of the light isotope [51]. As a result, organisms generally become enriched in the heavier isotope, i.e. have a higher δ value than their diet. Because this is a long-term process, stable isotope ratios in tissue reflect the diet over a period of weeks to months [52], [53]. In marine food webs there is generally an enrichment of approximately 3.0 to 5.0‰ in nitrogen (with a mean 2 ‰ for bird blood [46]) and 0.8‰ in carbon per trophic level [44], [51], [54]. Differences in nitrogen isotope ratios are frequently used to determine trophic level and diet composition (e.g. [55]−[57]). Depending on the tissue chosen, dietary information spanning different temporal scales can also be obtained [58]. In contrast to nitrogen, carbon isotope ratios differ more between terrestrial versus marine, inshore versus offshore and pelagic versus benthic food webs than by trophic level. Carbon can therefore be used to assess foraging location (reviewed in [59], [60]).

To assess individual consistency in the diet of the Dolphin Gull, we analyzed carbon and nitrogen stable isotopes of red blood cells from each of the individual studied. SIA of red blood cell samples integrates, for each individual, the foraging behavior over a period of several weeks (i.e. long-term variability and consistency) [45]. Of the two major constituents of whole blood, blood plasma and red blood cells, plasma has a much faster turn-over with a half-life of ca. three days, while red blood cells have a half-life of ca. 30 days and are therefore integrated over a much longer time [61].

The following diet samples seen to be consumed by Dolphin Gulls at New I. were included as stable isotope reference values: dried regurgitates of Imperial Shags obtained at the colonies on New I., Blue Mussel tissue from New I. and Beaver I., and krill *Euphasia* sp. washed up on a New I. beach. In previous studies [3], [62], we found that Imperial Shag regurgitates are mainly composed of fish, squid and lobster krill. Because the colonies in the region are not accessible, we were not able to obtain two other items seen to be consumed by Dolphin Gulls from New Island: Rock Shag regurgitates and Sea Lion feces. However, in Patagonia where Imperial and Rock shags also occur sympatrically, they feed on a similar trophic level [20]. Sea Lions and Gentoo Penguins also take similar prey [16], [25], therefore we consider the regurgitates of Imperial Shags included in the present study representative of the prey taken by the Dolphin Gull at the different seabird and mammal colonies.

Samples of whole krill and Blue Mussel tissue (without shells) were lipid extracted in a Soxhlet apparatus using chloroform:methanol for at least 6 hours until all lipids were extracted, the liquid no longer colored by any remaining lipids. The crustaceans were acid-washed to remove carbonate, 3.8 w/w % hydrochloric acid being slowly added until no further CO2-gas was formed. The remaining tissue was cleaned with de-ionized water. Afterwards, all samples were dried at 60°C to constant mass, for at least one day. Blood samples were centrifuged and red blood cells were dried at 38°C in an oven for further analysis. Aliquots of around 0.7 mg of each dry sample were weighed into tin capsules.

Stable isotope analyses were carried out at the Leibniz Institute for Zoo and Wildlife Research, Berlin, Germany. Carbon and nitrogen isotope ratios were measured simultaneously by continuous-flow isotope ratio mass spectrometry using a Flash Elemental Analyser (Thermo Finnigan, Bremen, Germany) linked to a Delta V Advantage Isotope Ratio Mass Spectrometer (Thermo Finnigan, Bremen, Germany). Two laboratory standards were analyzed for every 10 unknown samples, allowing any instrument drift over a typical 14 hour run to be corrected. Stable isotope ratios were expressed in δ notation as parts per thousand (‰) deviation from the international standards V-Pee dee belemnite (carbon) and AIR (nitrogen), according to the following equation δ X = [(R_sample_/R_standard_) –1] ×1000 where×is ^15^N or ^13^C and R is the corresponding ratio ^15^N/^14^N or ^13^C/^12^C. Based on internal standards (N = 165, tyrosin; Roth, Germany), the analytical precision (±1 SD) equaled ±0.16‰ and ±0.29‰ for δ^15^N and δ^13^C, respectively.

### Stable Isotope Data Analyses and Mixing Model

To estimate diet compositions based on stable isotope values, we applied a Bayesian model in SIAR 4.0 (Stable Isotope Analysis in R) [63] that runs under the free software R [64]. This model allows the incorporation of sources of uncertainty, in particular the variability in isotope signatures of prey species [47], [65]. SIAR uses Markov Chain Monte Carlo modeling, taking data on animal isotopes and fitting a Bayesian model to their dietary habits based upon a Gaussian likelihood with a Dirichlet prior mixture on the mean. The model assumes that each target value (i.e. the stable isotope data of each individual) comes from a Gaussian distribution with an unknown mean and SD. The structure of the mean is a weighted combination of the food sources’ isotopic values. The SD depends on the uncertainty around the fractionation corrections and the natural variability between target individuals within a defined group (in this case, males and females). We used the standard setting (20,000 iterations), and the following mean isotopic discrimination rates for diet-blood in birds reviewed in [41]: δ^15^N = 2‰, δ^13^C = 0.4‰. SD was set to 0.6 for δ^15^N and 0.03 for δ^13^C (e.g. [39]. We included regurgitates of Imperial Shags, Blue Mussel tissue, and krill as sources in the mixing model. The reference prey items were collected in the same breeding season of the logger study.

### Ethics Statement

This study was approved by the Falkland Islands Government (Environmental Planning Office) through the Research License R13/2008. The New Island Conservation Trust for permission to work on New Island. Extreme care was taken to minimize stress to the captured adults and to protect eggs from potential predators. Handling time was kept to a minimum, mostly less than 15 minutes and always less than 20 minutes. The head was covered during handling in order to minimize adult stress. During this procedure the birds remained relatively calm and no significant signs of stress were detected. Blood sampling had no detectable adverse effects. Potential impact of logger attachment is evaluated in the following analyses.

## Results

### Spatial and Temporal Data

In January 2009, tagged Dolphin Gulls breeding at New I. (Falkland Islands/Islas Malvinas, South-western Atlantic Ocean), foraged at several nearby islands in the southwest of the archipelago (Fig. 1). One thousand and forty-nine (1,049) GPS fixes were recorded during 66 foraging trips of eight incubating females and eight incubating males (Fig. 1). Individual data for each of the 16 instrumented Dolphin Gulls is shown in separate maps in the (File S2, Figs. S2− S17). The length of the foraging trips ranged from 3 to 41 kilometers, lasting from 1.5 to 15.5 hours, and reaching a maximum distance of 18 kilometers from the colony at New I. (Table 1). Individuals flew to their preferred foraging places one to three times per day (Table 1). Foraging trips took place mainly during the day hours (*n* = 62), except in the case of four individuals that performed one overnight foraging trip each. No differences in foraging trip parameters were observed between females and males (Table 1; see also File S2, Figs. S2− S17) despite strong sex differences in body mass (females, mean 529±46.3 g, range 430−590 g, *n* = 10; males, mean 611±23.3 g, range 570−650 g, *n* = 10; *t* = -5.0, *df* 18, *P* = <0.001).

### Kernel Distribution

**Figure 2:**
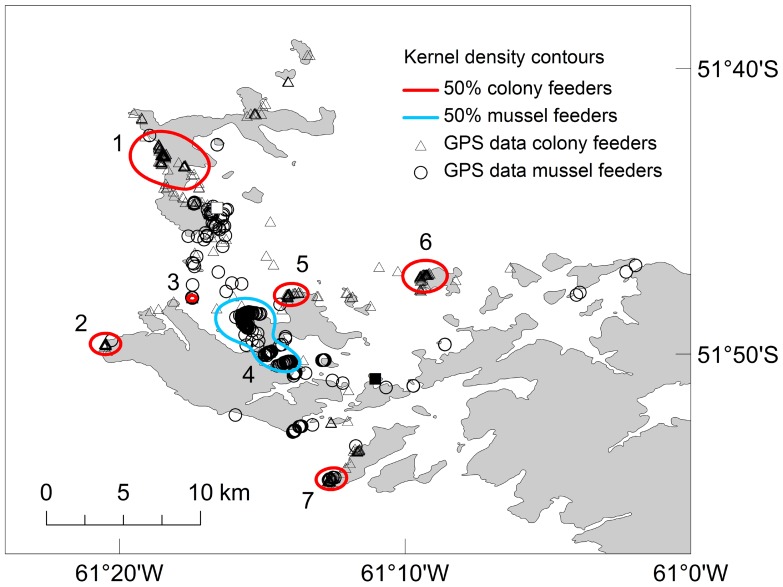
Kernel density analyses of GPS data of Dolphin Gulls *Leucophaeus scoresbii.* The 50% kernel density distribution of tagged dolphin gulls that repeatedly attended seabird and seal colonies (denoted as ‘colony feeders’; sites 1–3 and 5–7) are marked red. The 50% kernel density distribution of birds that repeatedly attended mussel beds (denoted as ‘mussel feeders’; site 4) are marked light blue. GPS locations of colony feeders are marked with triangles, while those of mussel feeders are marked with circles. Mussels, seals and seabirds present per site: site 1) Imperial Shags *Leucocarbo atriceps*, Rockhopper Penguins *Eudyptes chrysocome*, Black-browed Albatrosses *Thalassarche melanophris*; site 2) Imperial and Rock shags, Fur Seals *Arctocephalus australis*; site 3) Rock Shags; site 4) Blue Mussel *Mytilus edulis chilensis*; site 5) Imperial and Rock shags; site 6) Imperial and Rock shags, Southern Giant-petrels *Macronectes giganteus*; site 7) Imperial and Rock shags. The Dolphin Gull colony at New I. is indicated with a white square. A second Dolphin Gull colony in the region is marked with a black square.

Kernel density analysis of GPS data indicated two major foraging strategies in Dolphin Gulls from New Island (for 50, 60, 70, 80, 90 and 95% density contour areas see File S3, Figs. S18, S19). The 50% kernel distribution of tagged Dolphin Gulls detected 7 major foraging sites that individuals attended repeatedly: either an important Blue Mussel site in the area of Beaver Harbour (hereafter ‘mussel feeders’; site 4 in Fig. 2; File S1, Fig. S1, and File S3, Fig. S19) or several seabird and seal colonies on New I. and neighboring Beaver I. (hereafter ‘colony feeders’; sites 1–3 and 5–7 in Fig. 2; File S1, Fig. S1, and File S3, Fig. S18) during a week of tracking. The seabird colonies consisted mainly of Imperial Shags (sites 1, 2, 5, 6, and 7 in Fig. 2) and Rock Shags *Phalacrocorax magellanicus* (sites 2, 3, 5, 6, and 7 in Fig. 2). A smaller number of positional data were recorded in colonies of Rockhopper Penguins (site 1 in Fig. 2), Gentoo Penguins (Fig. 2, and File S1, Fig. S1), Black-browed Albatrosses (site 1 in Fig. 2), and Southern Giant-petrels (site 6 in Fig. 2), and Fur Seals (site 2 in Fig. 2).

No differences in foraging trip parameters were observed between mussel feeders and colony feeders (Table 1; File S2, Figs. S2− S17). However, we observed a significant difference in the adult body mass between mussel feeders and colony feeders (Table 2). Females foraging in mussel beds were significantly heavier than those attending seabird and seal colonies (Table 2). Overall males were heavier than females (Table 2).

**Table 2:** Sources of variation in adult body weight of Dolphin Gulls *Leucophaeus scoresbii*.

Gender	Foraging type			Mean	SD	*N*
Females	Colony feeders			526.0	18.2	5
	Mussel feeders			576.7	15.3	3
Males	Colony feeders			608.0	23.9	5
	Mussel feeders			603.3	20.8	3
**Type III Sum of Squares**			***df***	***F***	***P***	***η*** **^2^**
Foraging type		1983.750	1	4.8	0.048	0.524
Gender		11070.417	1	26.9	<0.001	0.997
Foraging type × Gender		2870.417	1	7.0	0.021	0.680
Error		4933.333	12			

General Linear Models (GLMs) based on Type III Sum of Squares were carried out with adult body weight at logger deployment as dependent variable and foraging type and gender as fixed factors. As a measure of effect size partial Eta-squared values (*η* ^2^) were included, i.e. the proportion of the effect+error variance that is attributable to the effect. The sums of the *η* ^2^ values are not-additive (e.g. http://web.uccs.edu/lbecker/SPSS/glm_effectsize.htm).

### Stable Isotope Analysis

**Figure 3:**
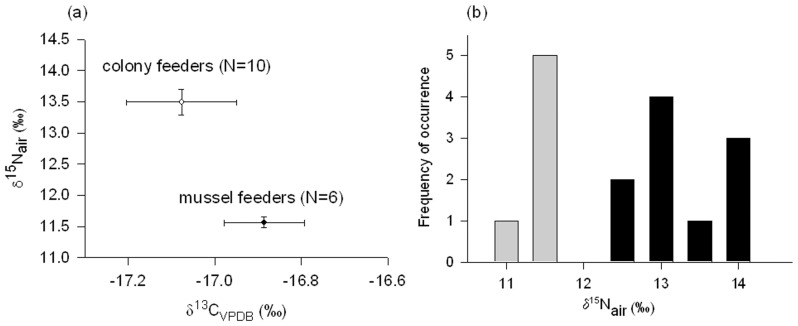
Stable isotope ratios in red blood cells of Dolphin Gulls *Leucophaeus scoresbii.* Isotope values (means and standard errors) correspond to blood samples taken during recapture of the birds and removal of the GPS logger during the breeding season. (a) Carbon and nitrogen isotope values of ‘mussel feeders’ (site 4 in Fig. 2), and ‘colony feeders’ (sites 1–3 and 5–7 in Fig. 2), (b) Distribution of nitrogen stable isotope values, with grey bars corresponding to 6 mussel feeders, and black bars corresponding to 10 colony feeders.

We compared the stable isotope values of red blood cells of two groups detected by GPS logging. The two groups (6 mussel feeders and 10 colony feeders) differed in their δ^15^N (Mann-Whitney Rank Sum Test, *U* = 21, *P* = 0.001; Fig. 3), but not in their δ^13^C (*t* = -1.1, *df* 14, *P* = 0.31; Fig. 3a). The clustering of δ^15^N values according to feeding areas (Fig. 3b) corresponded to strong δ^15^N differences between mussels (range 7.2−9.6‰) and Imperial Shag diet regurgitates (range 11.2−14.2‰: *t* = 11.4, *df* 17, *P*<0.001).

**Figure 4:**
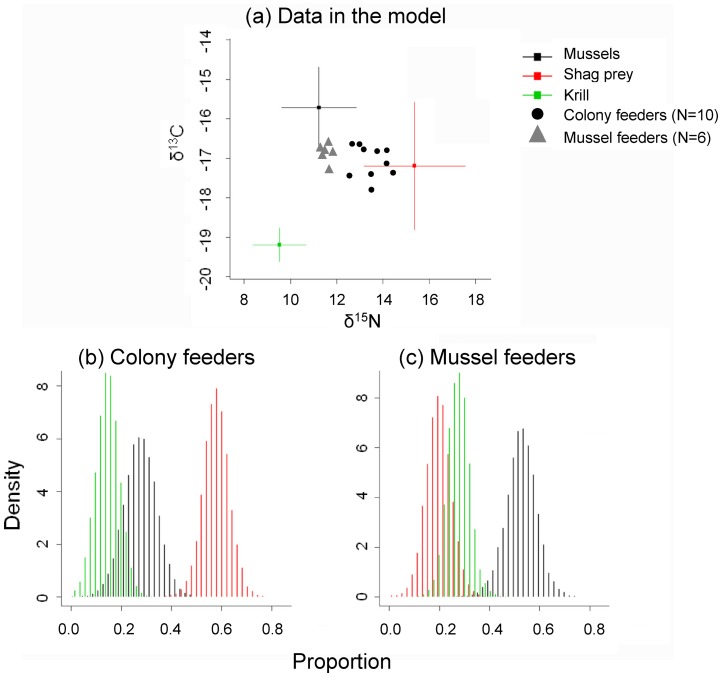
Diet compositions of Dolphin Gulls *Leucophaeus scoresbii* based on stable isotope values. Food type contributions according to a Bayesian model in SIAR 4.0 (Stable Isotope Analysis in R), based on red blood cell stable isotope values of tagged birds (a). Density plots show the contributions of main prey types to diet in colony feeders (b), and in mussels feeders (c).

The stable isotope values of the reference prey types (Blue Mussels, Imperial Shag regurgitates and krill) were well separated (Fig. 4a), suggesting their suitability as sources in a mixing model. The distributions resulting from the SIAR stable isotope mixing model (Fig. 4b, c) suggested that the individuals recorded mainly in Beaver Bay (File S1, Fig. S1) took 53% mussels (95% CI from 40 to 65%), 27% krill (95% CI from 18 to 36%), and 20% food obtained by scavenging from colonies (95% CI from 10 to 30%). Individuals recorded in other foraging sites fed much less on mussels (modeled mean 28%; 95% CI from 15 to 41%), but obtained much more food by scavenging from colonies (modeled mean 58%, 95% CI from 47 to 69%). Krill contributed 14% (95% CI from 5 to 23%). Dolphin Gulls find krill opportunistically on beaches where it is washed ashore, usually after storms (File S4, Fig. S23). These results indicate an individual consistency in foraging strategies over a period of several weeks, in line with our short term observations of foraging locations.

### Logger Impact on Bird Condition and Parameters of Breeding Success

Birds with loggers did not desert nests. In the studied nests (*n* = 20), the number of eggs did not vary significantly before and after deployment of the loggers (median before 2 eggs, median after 2.5 eggs, range 2−3 eggs, Mann-Whitney Rank Sum Test, *U* = 120, *P* = 0.86) or between nests with loggers and control nests (12 nests; *U* = 90, *P* = 0.43). Thus, the deployment of the loggers and the handling of the birds had no effect on clutch size and did not increase the risk of predation and/or nest failure. Additionally, no significant mass differences were detected in tagged birds before versus after logger deployment (before deployment mass, mean 570±35.5 g, range 520–620 g, *n* = 11; after, mean 548.2±56.7 g, range 450–630 g, *n* = 11; *t* = 2.1, *df* 10, *P* = 0.06).

## Discussion

We found that individual Dolphin Gulls specialized on particular feeding sites and diets, and the specialization was consistent over a range of several days (GPS loggers) as well as several weeks (stable isotope values).

According to [1], individual niche width depends on the diversity of available resources (‘ecological opportunity’), and resource abundance. When the number of individuals exceeds the available preferred resources, some individuals may act optimally by choosing an alternative niche, specializing in previously unused resources and thus reducing intra-specific competition. Such behavior may also be learned from adults and thus socially transmitted (e.g. [66]). Here we found that most studied Dolphin Gulls fed on the scraps of food dropped while larger seabirds such as Imperial Shags, Rock Shags or Gentoo Penguins fed their chicks (Fig. 3; see also Boulder Point in File S5, Table S1, and in File S4, Fig. S20). However, several individuals consistently foraged in large mussel beds (Fig. 3; see also site 4 in Fig. 2) by picking out Blue Mussels, which they opened by dropping from some height onto the rocks below (File S4, Figs. S21, S22). Dolphin Gulls were furthermore observed in Sea Lion colonies (see observations from Stick in the Mud I. in File S5, Table S1), but we recorded no individuals specialized in foraging at Sea Lion colonies, probably due to the sample size of instrumented Dolphin Gulls.

Gulls are generally classified as dietary generalist carnivores (e.g. [67]–[71]), but a similarly high degree of individual specialization for particular diets or foraging locations has been found in other gull species. Individual Herring Gulls *Larus argentatus* in Newfoundland were specialized in either Blue Mussels or in human refuse or in seabird prey [71]. A population of Western Gulls *Larus occidentalis* contained specialists foraging on fish or on human refuse [72]. Foraging area fidelity was observed in Black-legged Kittiwakes *Rissa tridactyla* as well as in Olrog’s Gull *Larus atlanticus* [73], [74]. This behavior may increase foraging efficiency, as birds can learn when and where to obtain prey, reducing the time required searching for feeding habitats [73], [74]. Foraging area fidelity is thus more likely to occur where prey is spatially predictable or concentrated [74]. But, which mechanisms could generate the observed specialization for particular feeding sites in Dolphin Gulls?

The present data did not support any differences based on gender or morphology (Tables 1 and 2). Although Dolphin Gull males are heavier than females (Table 2 and [16]), we detected no consistent differences in foraging between the sexes (Table 1). All calculated trip parameters were similar between females and males (Table 1). Also the proportion of males and females attending mussel beds or seabird and seal colonies was 1∶1. Consequently, the specialization in mussel feeders and colony feeders cannot be explained by sex-related differences.

It has been suggested that temporal pattern of food availability may influence individuals to take certain prey. For example, Dolphin Gulls at Punta Tombo are thought to be mainly associated with sea lions and cormorants, because cormorant food scraps and sea lion excrement are available throughout the day. In contrast, most Magellanic Penguins feed their chicks early and late in the day, and little food is available to Dolphin Gulls in the penguin colony at other times [15]. Likewise, in Herring Gulls the main food sources are available for different amounts of time and at different times each day [70]. However, in our study temporal restrictions are unlikely to explain the specialization pattern for several reasons: Firstly, the stable isotope data clearly showed that the individual specialization was maintained over several weeks prior to the study, including time before the onset of incubation duties. Secondly, patterns of food availability are not fixed (e.g. low tides that determine the availability of mussels do not occur at constant times of day). Furthermore, most foraging trips were long enough to include a low tide independent of the time of departure.

Similarly, other studies on seabirds showed that adults specialized in diet over long periods (e.g. [48], [49], [75], [76]), and even across several years of continued monitoring (e.g. [11]). For example, Brünnich’s Guillemots *Uria lomvia* specialize on a particular fish or crustacean species regardless of whether chick-provisioning and self-feeding [11]. The authors suggested that specialization likely represents learning and memorizing optimal feeding locations and behaviors. Optimal Foraging Theory [77] predicts that individuals prefer prey with a high energetic value per unit of search and handling time. The search and handling time for a given prey type, which thus largely determines the energy gain during foraging, may vary importantly among individuals, depending on their experience and learning. When the gain from foraging can be increased strongly by experience with a particular food source and foraging site, it may pay an individual to specialize in foraging at known sites and on known prey items. Different food items may require different, sometimes complex, acquiring or processing techniques so that a given individual may only be able to learn a limited number of them. This would limit the time available to individuals to learn new skills, driving them to specialize in known foraging techniques (e.g. [78]−[80]). Elaborate processing techniques seem to be particularly important to mussel-feeding in gulls, which requires particular complex skills: gulls have to time foraging with low tide, extract mussels, fly up and drop them on exposed rocks, and dive down immediately to eat their prey before being kleptoparasitized by other birds (see File S4, Figs. S20− S23). Among Herring Gulls, individuals that specialized in mussels were more successful breeders, with larger clutch sizes, higher hatching success, and more fledglings [71]. Our finding that females foraging at mussel beds were significantly heavier than those attending seabird colonies (Table 2) fits in well with this observation. Furthermore, feeding on mussels might be advantageous, as mussels are available year-round, independently of the timing of breeding of other seabirds and marine mammals.

Intraspecific competition for food may reduce the availability of the favored diet components, motivating individuals to expand their niche to less preferred items. If all individuals prefer the same resources but select different secondary resources, then as individuals expand their niches they will tend to diverge in their preferences (e.g. [1], [6], and references therein). In this context, socially dominant individuals will secure the best resources, while subordinates may be unable to access them. Likewise, older, more competitive and more experienced individuals may specialize in the most profitable resources, as they master the necessary acquiring or processing techniques, while less experienced individuals may be forced to specialize on less profitable food items (e.g. [71]).

### Concluding Remarks

Individual Dolphin Gulls at New I. repeatedly foraged at the same place, suggesting individuals have an affinity for particular foraging locations and diet types. This may increase foraging efficiency, reducing the time required searching for feeding habitats. Stable isotope analyses allowed us to establish that dietary individual specialization observed over several days was maintained for a prolonged time. The present data do not allow us to distinguish more experienced or older from younger breeding birds, or test for offspring imprinting. However, the fact that mussel feeding females were heavier suggests that this might be a particularly favorable strategy for individuals which have learned the complex behavioral pattern needed to open the mussels. It would now be interesting to determine whether one of the foraging specializations is advantageous in the long run or during specific seasons or years. A larger sample size will be necessary to achieve this, but the present data suggest that stable isotope data can provide the necessary information to distinguish strategies.

## Supporting Information

File S1 Figure S1 Mussel beds, seal and seabird colonies located in New Island and surrounding islands. **M**: Blue Mussel *Mytilus edulis chilensis* beds, **IS**: Imperial Shags *Leucocarbo atriceps*, **RS**: Rock Shags *Phalacrocorax magellanicus*, **RP**: Rockhopper Penguins *Eudyptes chrysocome*, **GP**: Gentoo Penguins *Pygoscelis papua*, **BBA**: Black-browed albatross *Thalassarche melanophris*, **SGP**: Southern Giant Petrels *Macronectes giganteus*, **FS**: Fur Seals *Arctocephalus australis*.
(PDF)

File S2 **Figure S2–S17.** Figure S2. GPS fixes of female Dolphin Gull number 682, a mussel feeder. Figure S3. GPS fixes of female Dolphin Gull number 683, a colony feeder. Figure S4. GPS fixes of female Dolphin Gull number 686, a mussel feeder. Figure S5. GPS fixes of female Dolphin Gull number 688, a colony feeder. Figure S6. GPS fixes of female Dolphin Gull number 689, a colony feeder. Figure S7. GPS fixes of female Dolphin Gull number 690, a mussel feeder. Figure S8. GPS fixes of female Dolphin Gull number 692, a colony feeder. Figure S9. GPS fixes of female Dolphin Gull number 696, a colony feeder. Figure S10. GPS fixes of male Dolphin Gull number 684, a colony feeder. Figure S11. GPS fixes of male Dolphin Gull number 685, a colony feeder. Figure S12. GPS fixes of male Dolphin Gull number 687, a colony feeder. Figure S13. GPS fixes of male Dolphin Gull number 691, a mussel feeder. Figure S14. GPS fixes of male Dolphin Gull number 693, a mussel feeder. Figure S15. GPS fixes of male Dolphin Gull number 695, a mussel feeder. Figure S16. GPS fixes of male Dolphin Gull number 697, a mussel feeder. Figure S17. GPS fixes of male Dolphin Gull number 700, a colony feeder.
(PDF)

File S3 **Figure S18–Figure S19.** Figure S18. Kernel density analyses of GPS data of Dolphin Gulls *Leucophaeus scoresbii*. The 50, 60, 70, 80, 90 and 95% density contour areas of tagged dolphin gulls that repeatedly attended seabird and seal colonies (denoted as ‘colony feeders’). GPS locations of colony feeders are marked with triangles. Mussels, seals and seabirds present per site: site 1) Imperial Shags *Leucocarbo atriceps*, Rockhopper Penguins *Eudyptes chrysocome*, Black-browed Albatrosses *Thalassarche melanophris*; site 2) Imperial Shags and Rock Shags *Phalacrocorax magellanicus*, Fur Seals *Arctocephalus australis*; site 3) Rock Shags; site 4) Blue Mussel *Mytilus edulis chilensis*; site 5) Imperial and Rock shags; site 6) Imperial and Rock shags, Southern Giant-petrels *Macronectes giganteus*; site 7) Imperial and Rock shags. The Dolphin Gull colony at New I. is indicated with a white square. A second Dolphin Gull colony in the region is marked with a black square. Figure S19. Kernel density analyses of GPS data of Dolphin Gulls *Leucophaeus scoresbii.* The 50, 60, 70, 80, 90 and 95% density contour areas of tagged dolphin gulls that repeatedly attended mussel beds (denoted as ‘mussel feeders’). GPS locations of mussel feeders are marked with circles. Mussels, seals and seabirds present per site: site 1) Imperial Shags *Leucocarbo atriceps*, Rockhopper Penguins *Eudyptes chrysocome*, Black-browed Albatrosses *Thalassarche melanophris*; site 2 Imperial Shags and Rock Shags *Phalacrocorax magellanicus*, Fur Seals *Arctocephalus australis*; site 3) Rock Shags; site 4) Blue Mussel *Mytilus edulis chilensis*; site 5) Imperial and Rock shags; site 6) Imperial and Rock shags, Southern Giant-petrels *Macronectes giganteus*; site 7) Imperial and Rock shags. The Dolphin Gull colony at New I. is indicated with a white square. A second Dolphin Gull colony in the region is marked with a black square.
(PDF)

File S4 **Figure S20–S23.** Figure S20. Feeding in seabird colonies. Most dolphin gulls *Leucophaeus scoresbii* (Traill, 1823) at New Island feed on the scraps of food dropped while larger seabirds such as imperial cormorants *Leucocarbo atriceps* (King, 1828) (upper picture) or gentoo penguins *Pygoscelis papua* (J. R. Forster, 1781) (lower picture) feed their chicks. Usually, dolphin gulls walk or fly close to a cormorant or penguin while feeding chicks. When the birds interrupt their feeding to attack the gull, they commonly drop some food, which is then picked up by the gull. Figure S21. Feeding in mussel beds. Dolphin gulls feed in mussel beds (upper left picture) by picking out blue mussels (*Mytilus edulis chilensis*), which they open by dropping them from some height unto the rocks below (lower picture). The mussel shells with holes are regularly found as a result (upper right picture). Figure S22. Feeding in mussel beds Dense beds or mats of Blue Mussel *Mytilus edulis chilensis* are common in the Falkland Islands/Islas Malvinas but largely confined to sheltered bays and inlets. A particularly large Blue Mussel bed is located in Beaver Harbour, on the east side of Beaver Island (pictures below; see also Fig. 1). Figure S23. Additional food source. Occasionally, dolphin gulls picked up krill larvae or other invertebrates that washed ashore after storms in the intertidal zone.
(PDF)

File S5 **Table S1.** Location, identity, and size of seabird and mammal colonies visited by the Dolphin Gull *Leucophaeus scoresbii* from New Island. Places were surveyed from a sailing boat in December 2010.
(XLSX)
