# Supplementary material for: Distribution Patterns Predict Individual Specialization in the Diet of Dolphin Gulls
Source: PLoS One. 2013 Jul 2;8(7):e67714. doi: 10.1371/journal.pone.0067714 (PMC3699636; doi:10.1371/journal.pone.0067714)
Supplement: File S1 — Figure S1 Mussel beds, seal and seabird colonies located in New Island and surrounding islands. M: Blue Mussel Mytilus edulis chilensis beds, IS: Imperial Shags Leucocarbo atriceps, RS: Rock Shags Phalacrocorax magellanicus, RP: Rockhopper Penguins Eudyptes chrysocome, GP: Gentoo Penguins Pygoscelis papua, BBA: Black-browed albatross Thalassarche melanophris, SGP: Southern Giant Petrels Macronectes giganteus, FS: Fur Seals Arctocephalus australis. (PDF) [file pone.0067714.s001.pdf]

Supporting information to:

Distribution patterns predict individual specialization in the diet of Dolphin Gulls

Juan F. Masello, Martin Wikelski, Christian C. Voigt, Petra Quillfeldt

Content:

Figure S1. Mussel beds, seal and seabird colonies located in New Island and surrounding islands. M: Blue Mussel *Mytilus edulis chilensis* beds, **IS**: Imperial Shags *Leucocarbo atriceps*, **RS**: Rock Shags *Phalacrocorax magellanicus*, **RP**: Rockhopper Penguins *Eudyptes chrysocome*, **GP**: Gentoo Penguins *Pygoscelis papua*, **BBA**: Black-browed albatross *Thalassarche melanophris*, **SGP**: Southern Giant Petrels *Macronectes giganteus*, **FS**: Fur Seals *Arctocephalus australis*.

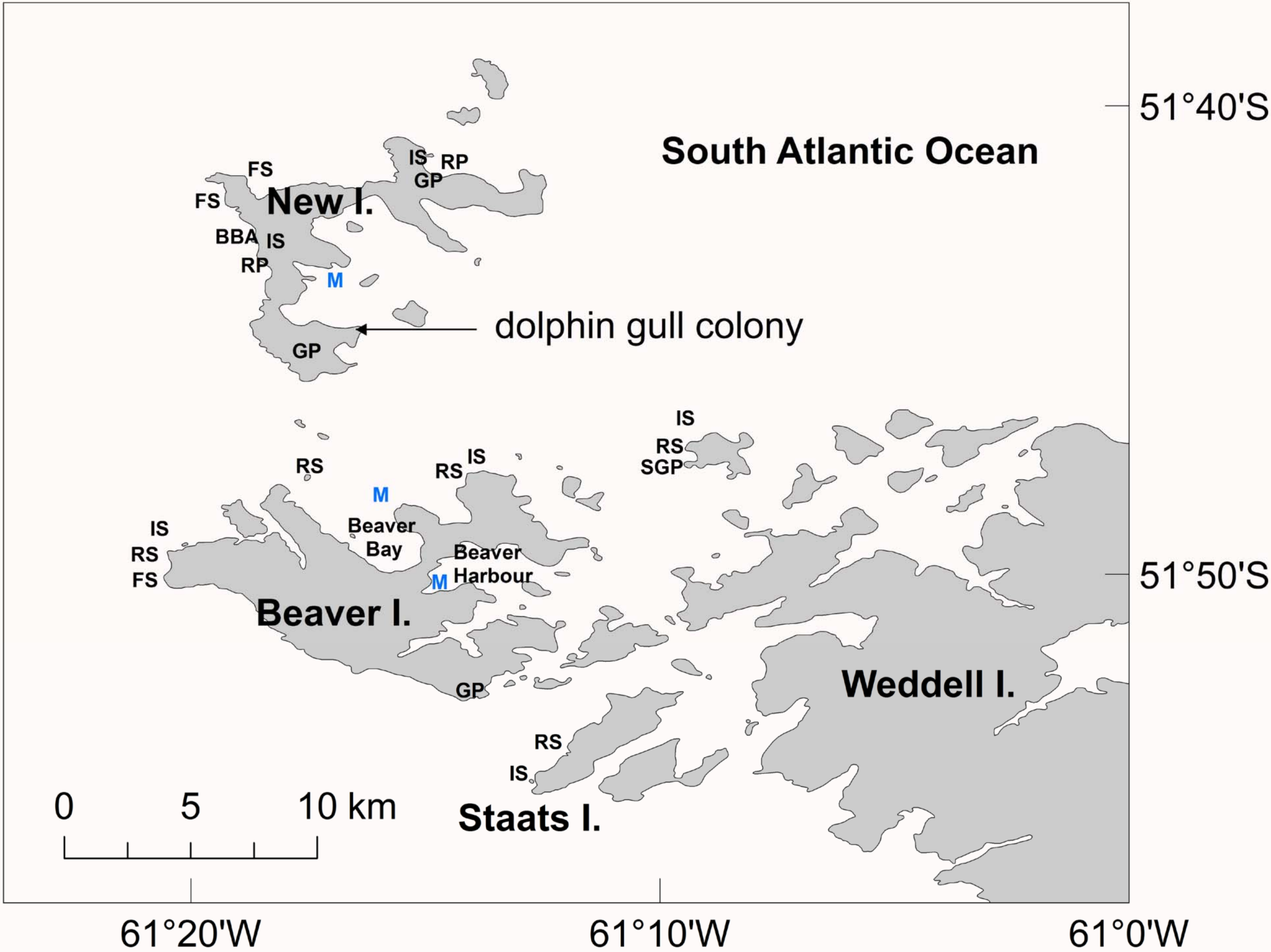

51°40'S
South Atlantic Ocean
FS
IS
RP
GP
FS
New I.
BBA
IS
RP
M
dolphin gull colony
GP
IS
RS
SGP
RS
IS
RS
M
IS
RS
FS
Beaver Bay
Beaver Harbour
M
51°50'S
Beaver I.
GP
Weddell I.
RS
IS
0
5
10 km
Staats I.
61°20'W
61°10'W
61°0'W
