# Supplementary material for: Distribution Patterns Predict Individual Specialization in the Diet of Dolphin Gulls
Source: PLoS One. 2013 Jul 2;8(7):e67714. doi: 10.1371/journal.pone.0067714 (PMC3699636; doi:10.1371/journal.pone.0067714)
Supplement: File S2 — Figure S2–S17. Figure S2. GPS fixes of female Dolphin Gull number 682, a mussel feeder. Figure S3. GPS fixes of female Dolphin Gull number 683, a colony feeder. Figure S4. GPS fixes of female Dolphin Gull number 686, a mussel feeder. Figure S5. GPS fixes of female Dolphin Gull number 688, a colony feeder. Figure S6. GPS fixes of female Dolphin Gull number 689, a colony feeder. Figure S7. GPS fixes of female Dolphin Gull number 690, a mussel feeder. Figure S8. GPS fixes of female Dolphin Gull number 692, a colony feeder. Figure S9. GPS fixes of female Dolphin Gull number 696, a colony feeder. Figure S10. GPS fixes of male Dolphin Gull number 684, a colony feeder. Figure S11. GPS fixes of male Dolphin Gull number 685, a colony feeder. Figure S12. GPS fixes of male Dolphin Gull number 687, a colony feeder. Figure S13. GPS fixes of male Dolphin Gull number 691, a mussel feeder. Figure S14. GPS fixes of male Dolphin Gull number 693, a mussel feeder. Figure S15. GPS fixes of male Dolphin Gull number 695, a mussel feeder. Figure S16. GPS fixes of male Dolphin Gull number 697, a mussel feeder. Figure S17. GPS fixes of male Dolphin Gull number 700, a colony feeder. (PDF) [file pone.0067714.s002.pdf]

Supporting information to:

Distribution patterns predict individual specialization in the diet of Dolphin Gulls

Juan F. Masello, Martin Wikelski, Christian C. Voigt, Petra Quillfeldt

Content

GPS fixes of the 16 studied Dolphin Gulls *Leucophaeus scoresbii* is shown in separate maps.

Figure S2. GPS fixes of female Dolphin Gull number 682, a mussel feeder.
Figure S3. GPS fixes of female Dolphin Gull number 683, a colony feeder.
Figure S4. GPS fixes of female Dolphin Gull number 686, a mussel feeder.
Figure S5. GPS fixes of female Dolphin Gull number 688, a colony feeder.
Figure S6. GPS fixes of female Dolphin Gull number 689, a colony feeder.
Figure S7. GPS fixes of female Dolphin Gull number 690, a mussel feeder.
Figure S8. GPS fixes of female Dolphin Gull number 692, a colony feeder.
Figure S9. GPS fixes of female Dolphin Gull number 696, a colony feeder.
Figure S10. GPS fixes of male Dolphin Gull number 684, a colony feeder.
Figure S11. GPS fixes of male Dolphin Gull number 685, a colony feeder.
Figure S12. GPS fixes of male Dolphin Gull number 687, a colony feeder.
Figure S13. GPS fixes of male Dolphin Gull number 691, a mussel feeder.
Figure S14. GPS fixes of male Dolphin Gull number 693, a mussel feeder.
Figure S15. GPS fixes of male Dolphin Gull number 695, a mussel feeder.
Figure S16. GPS fixes of male Dolphin Gull number 697, a mussel feeder.
Figure S17. GPS fixes of male Dolphin Gull number 700, a colony feeder.

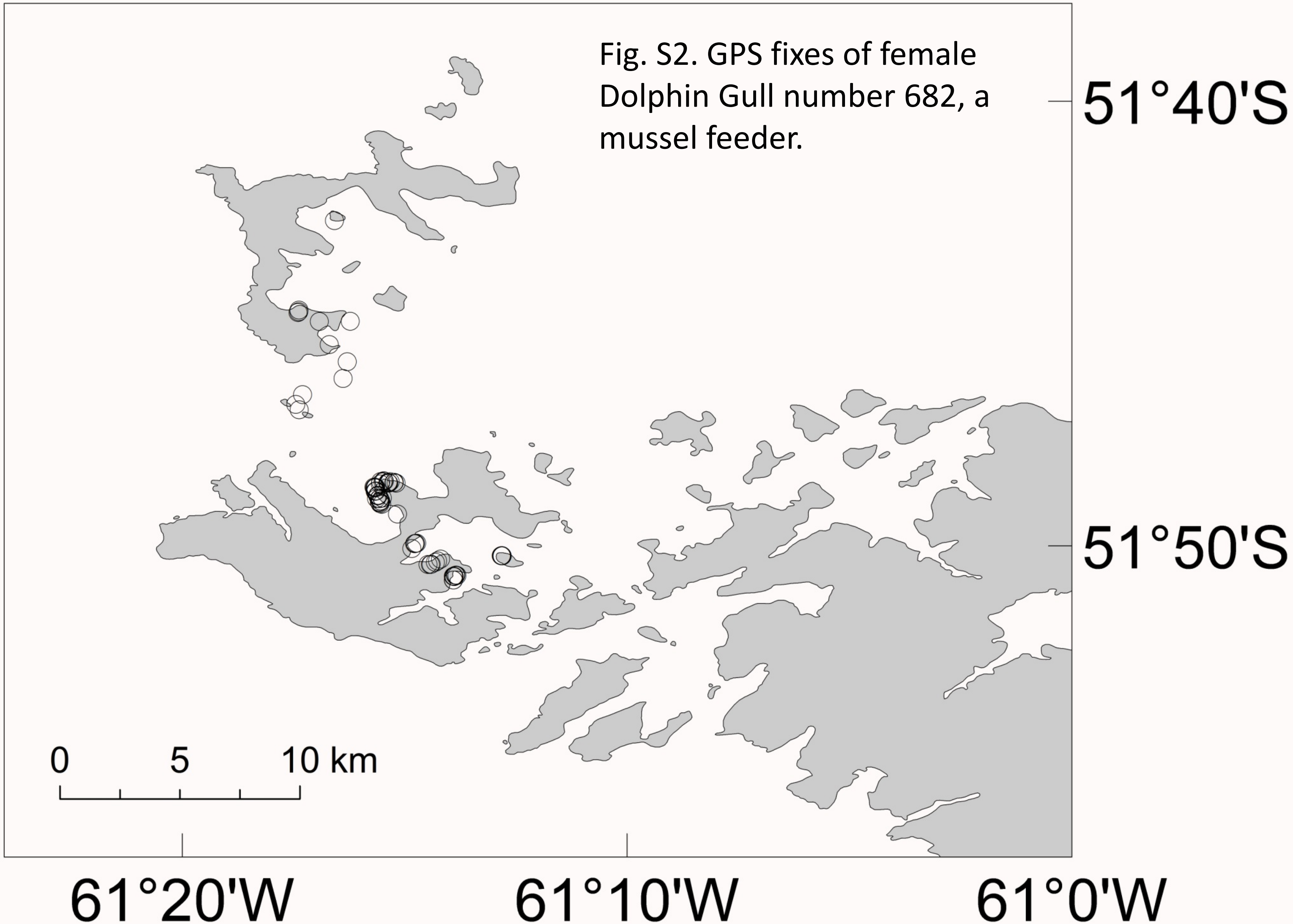

Fig. S2. GPS fixes of female Dolphin Gull number 682, a mussel feeder.

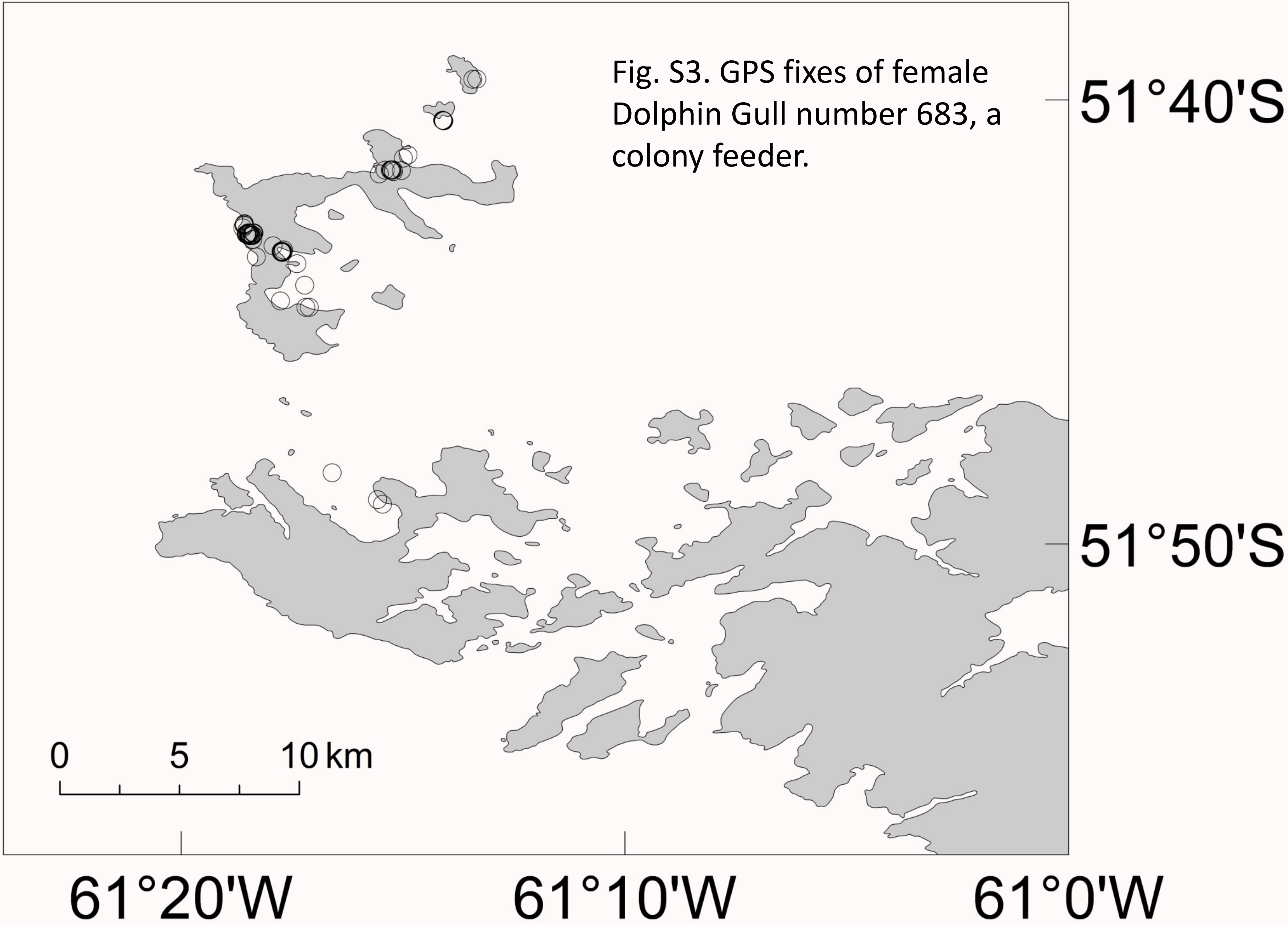

Fig. S3. GPS fixes of female Dolphin Gull number 683, a colony feeder.

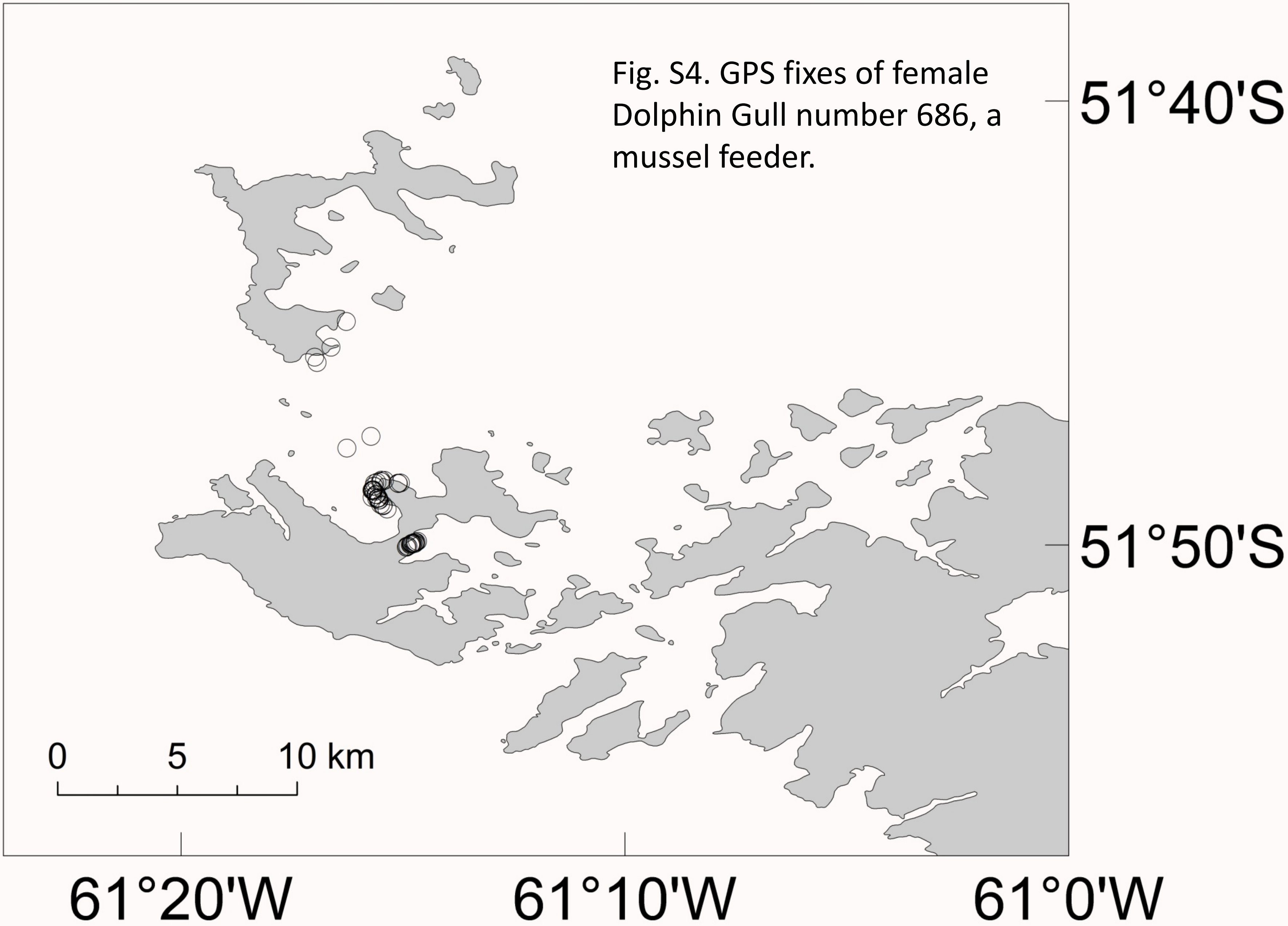

Fig. S4. GPS fixes of female Dolphin Gull number 686, a mussel feeder.

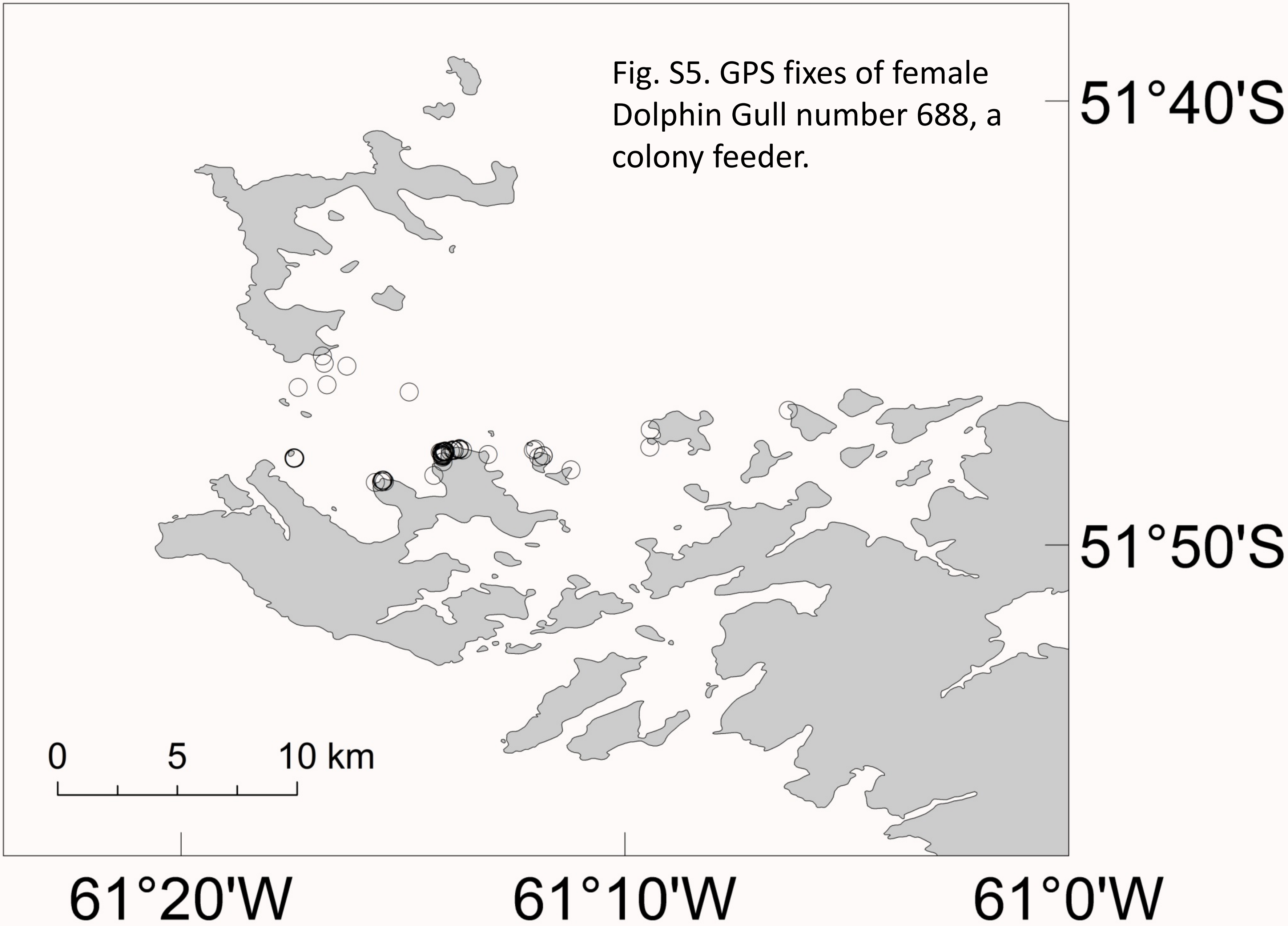

Fig. S5. GPS fixes of female Dolphin Gull number 688, a colony feeder.

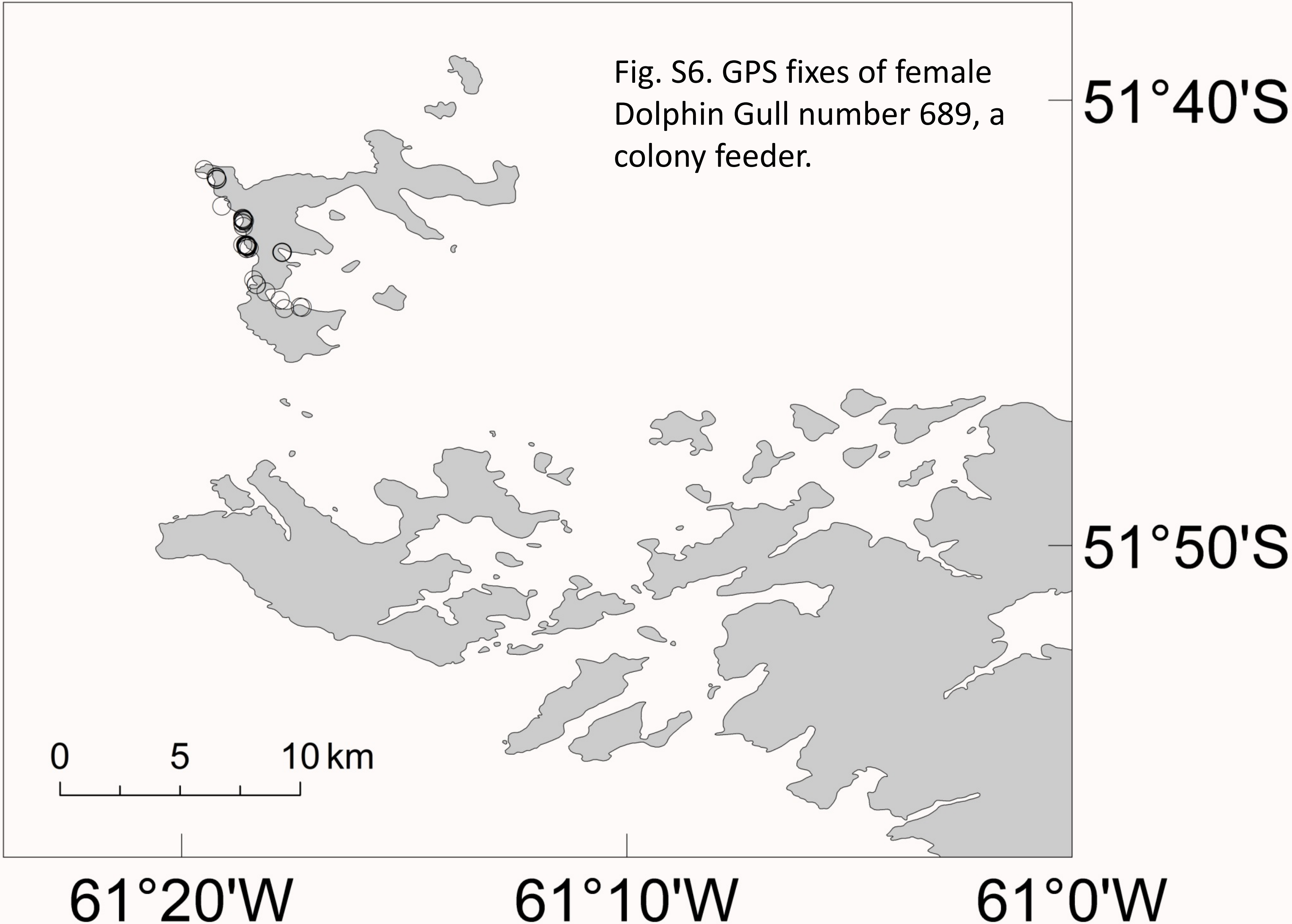

Fig. S6. GPS fixes of female Dolphin Gull number 689, a colony feeder.

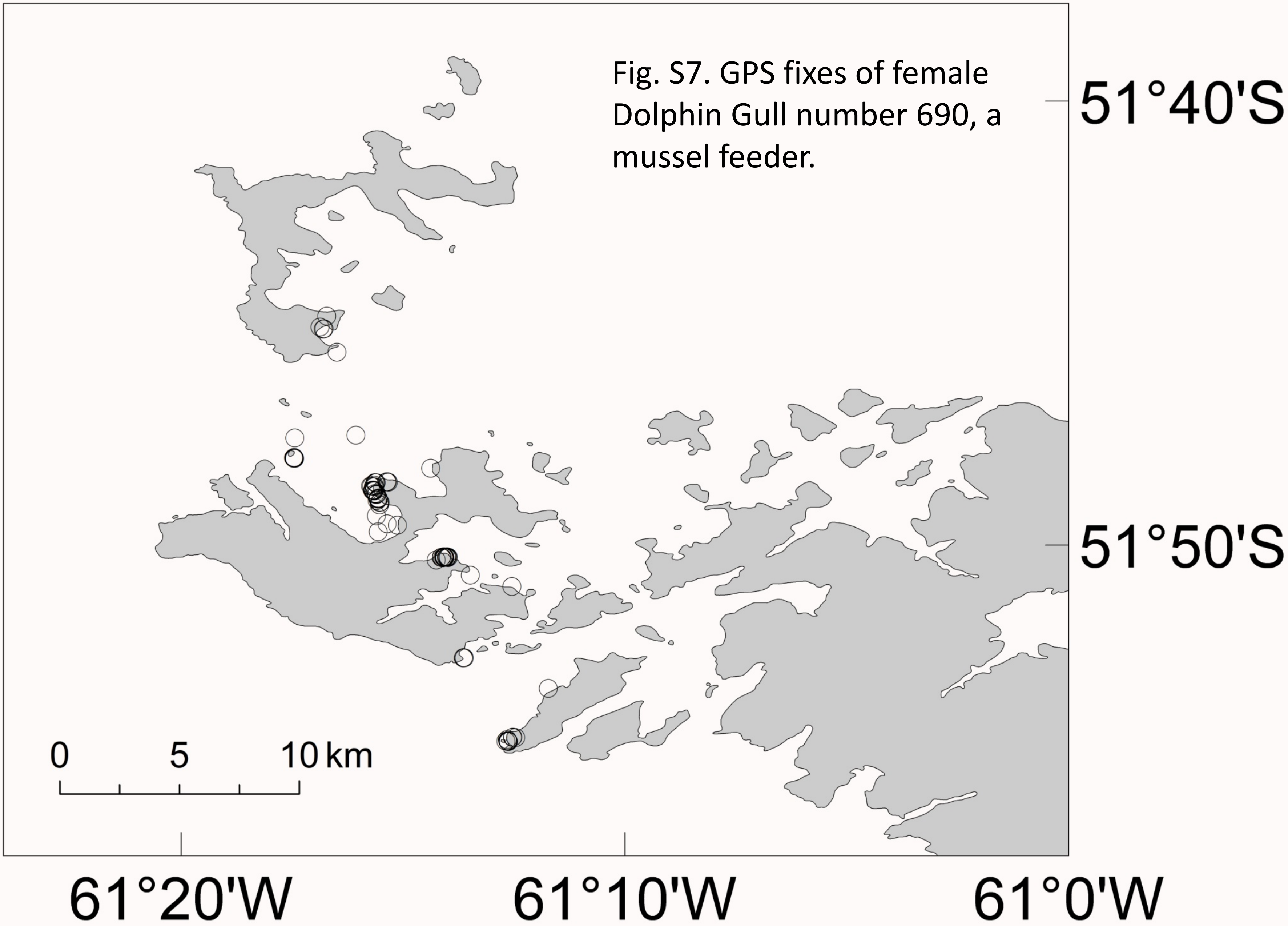

Fig. S7. GPS fixes of female Dolphin Gull number 690, a mussel feeder.

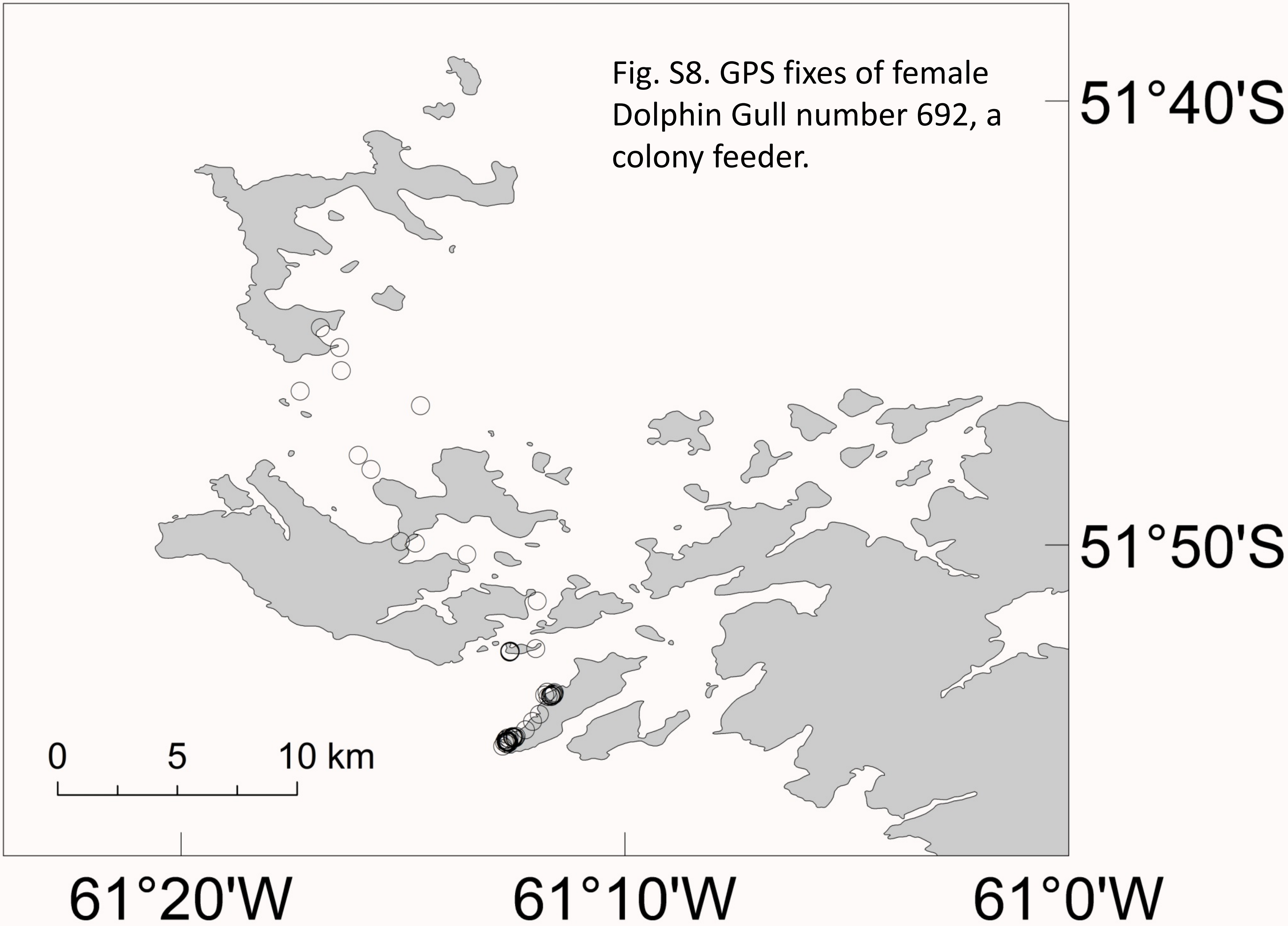

Fig. S8. GPS fixes of female Dolphin Gull number 692, a colony feeder.

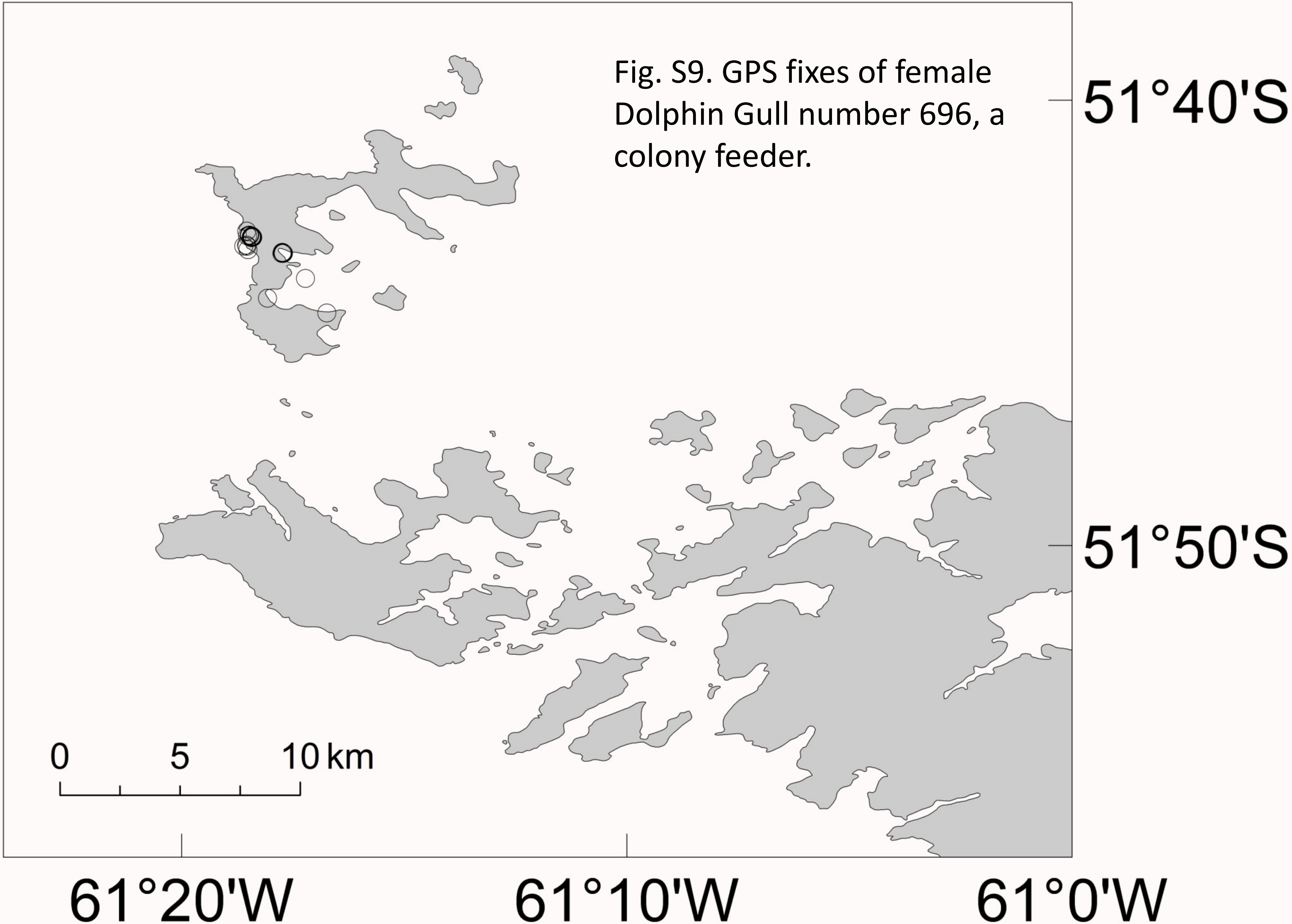

Fig. S9. GPS fixes of female Dolphin Gull number 696, a colony feeder.

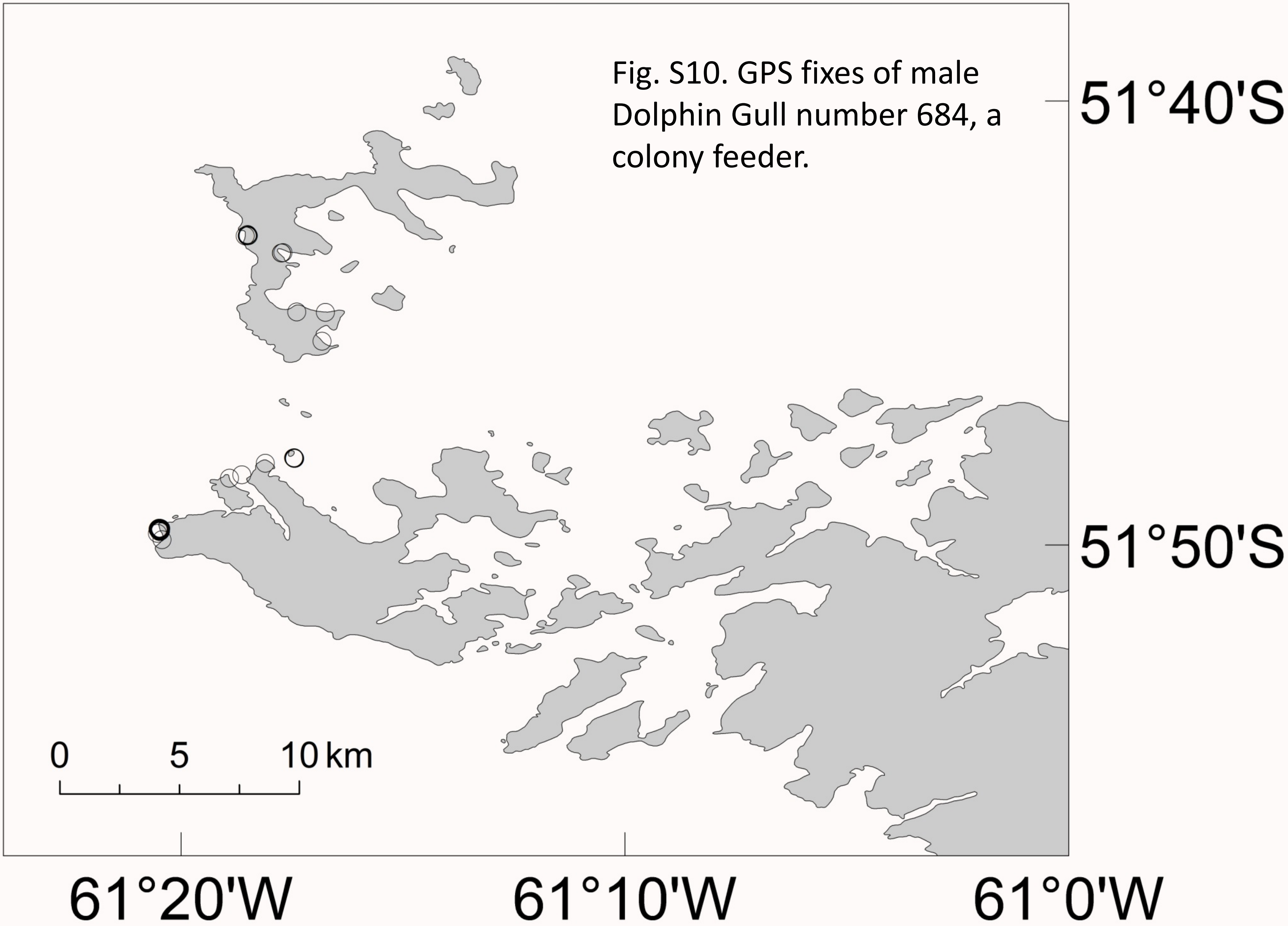

Fig. S10. GPS fixes of male Dolphin Gull number 684, a colony feeder.

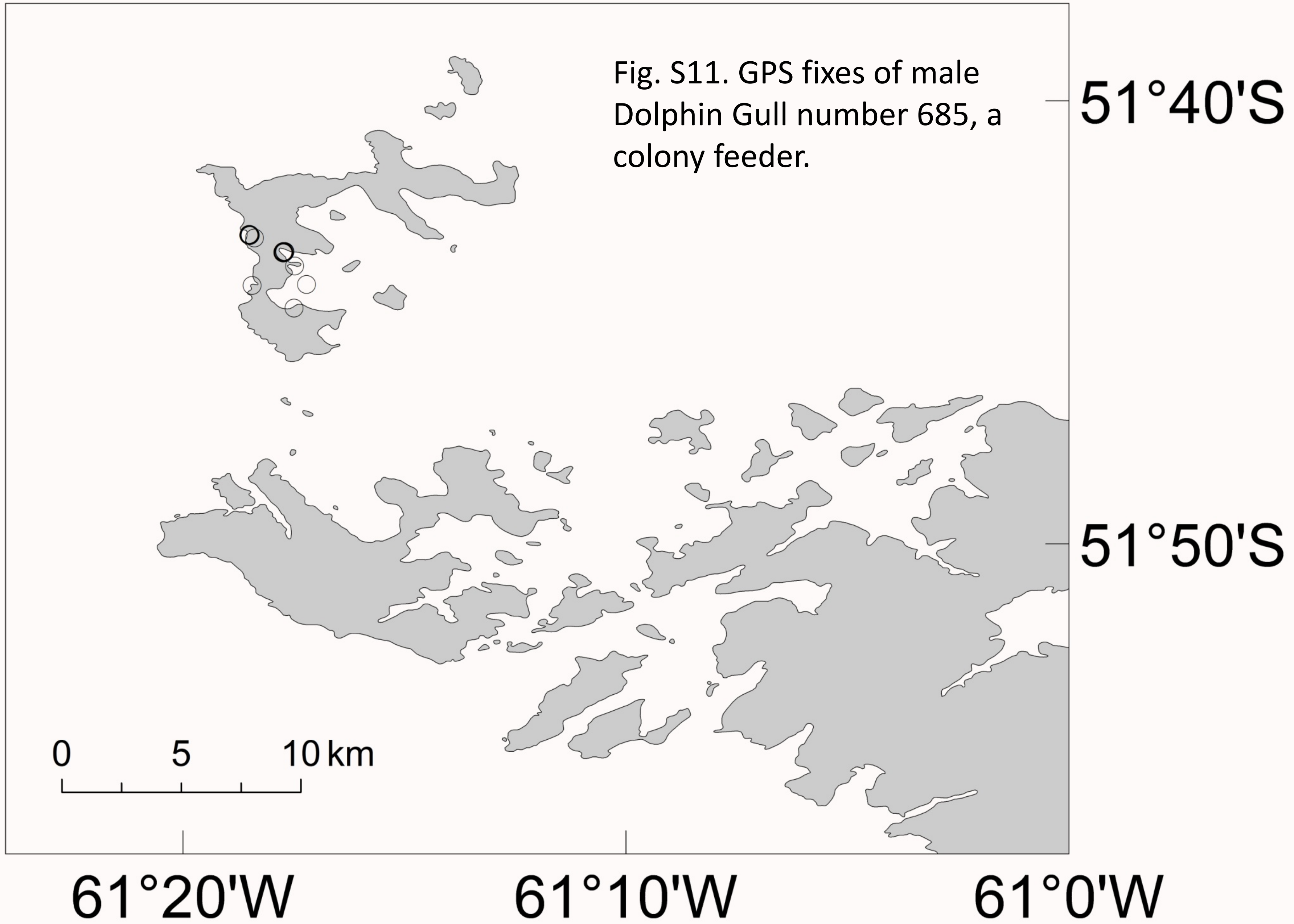

Fig. S11. GPS fixes of male Dolphin Gull number 685, a colony feeder.

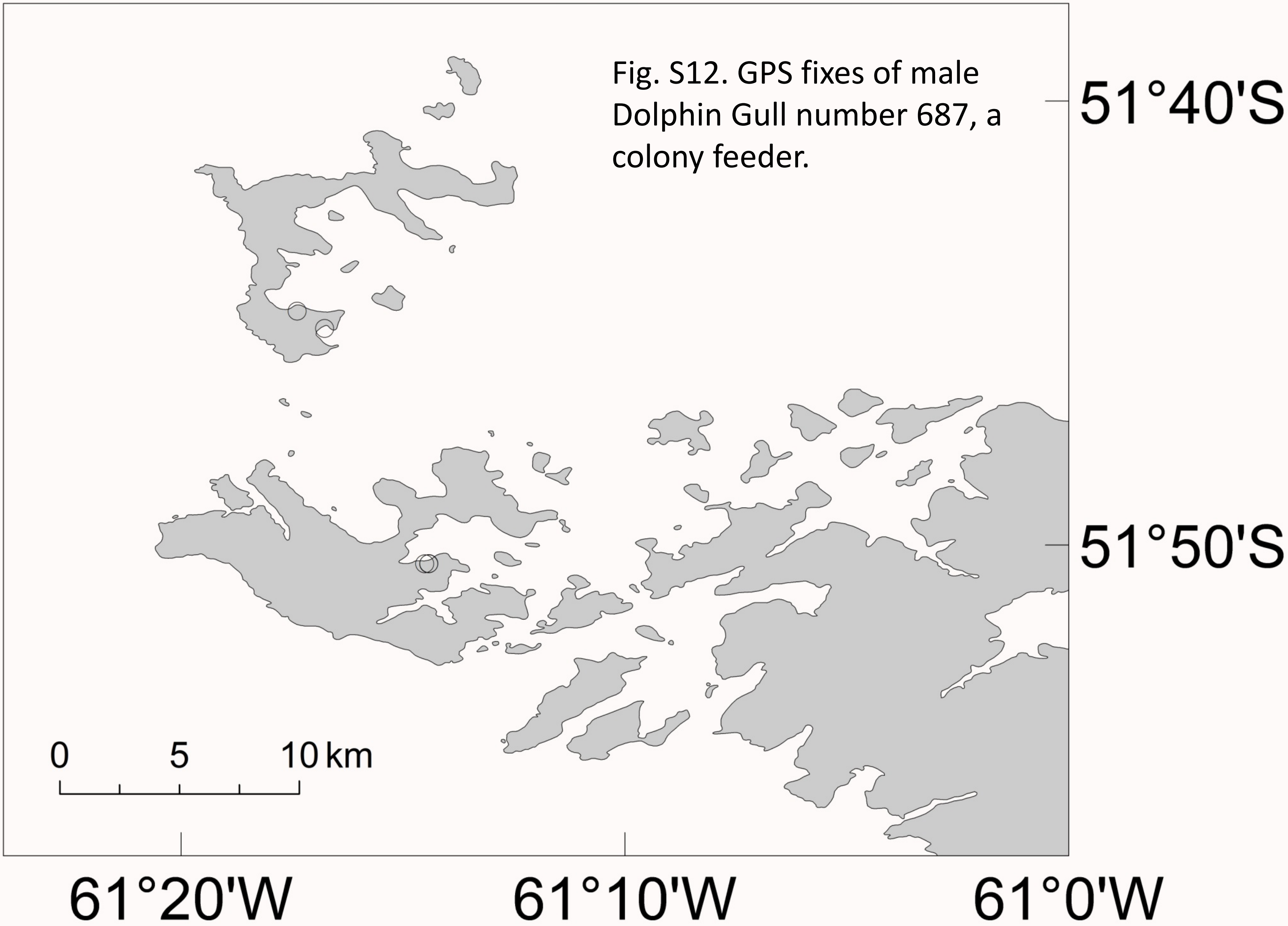
Fig. S12. GPS fixes of male Dolphin Gull number 687, a colony feeder.
51°40'S
51°50'S
0
5
10 km
61°20'W
61°10'W
61°0'W

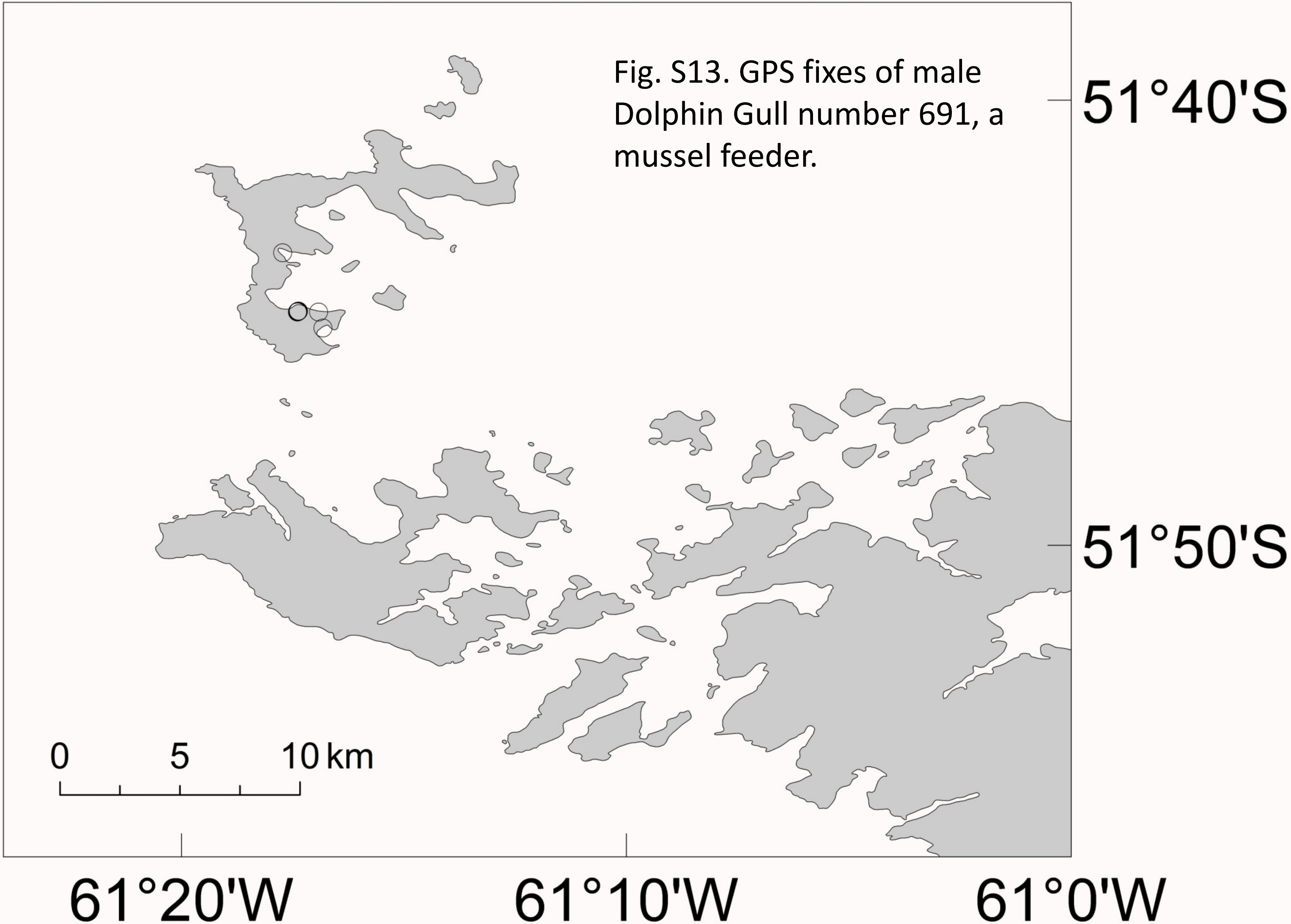

Fig. S13. GPS fixes of male Dolphin Gull number 691, a mussel feeder.

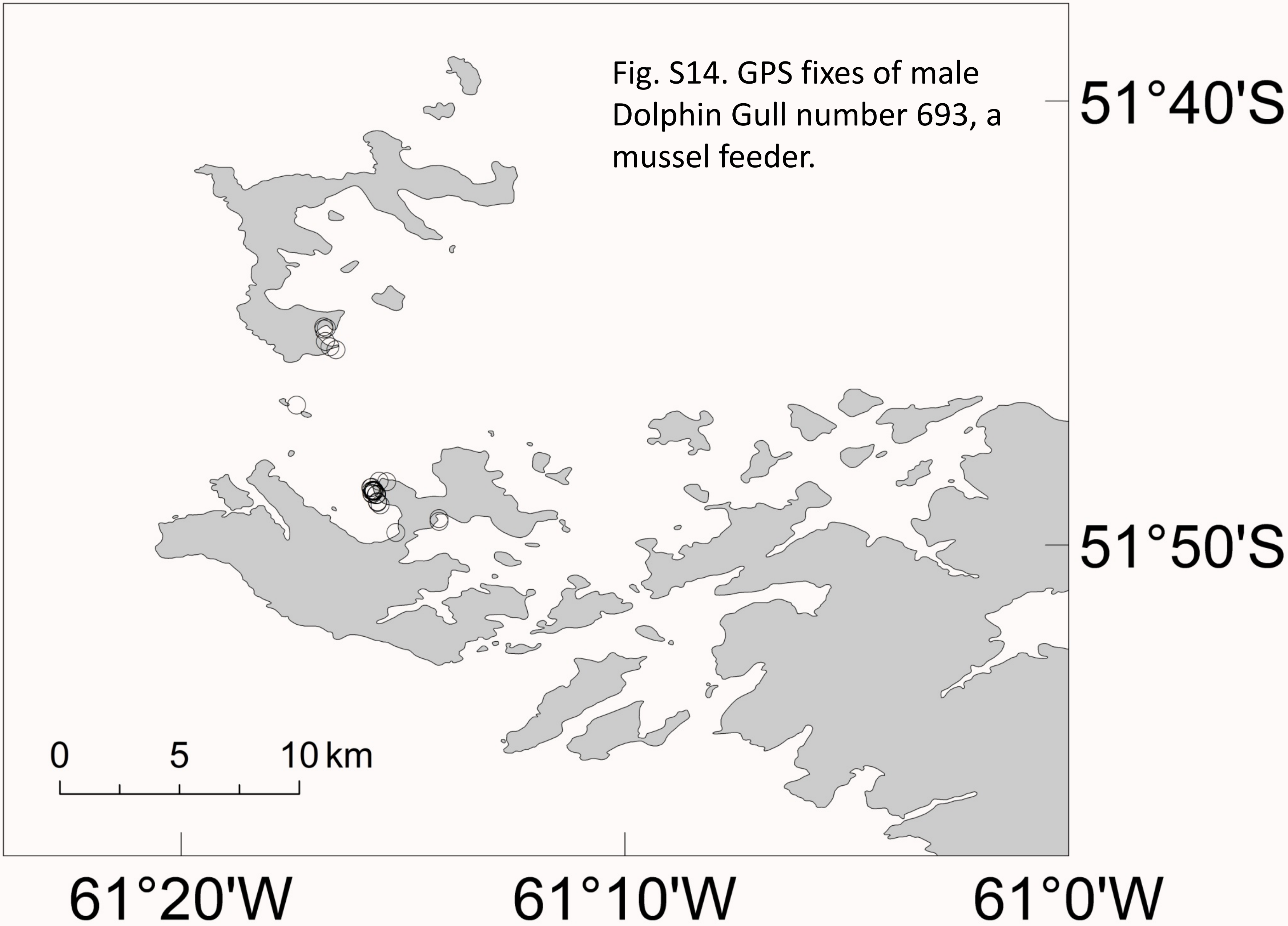

Fig. S14. GPS fixes of male Dolphin Gull number 693, a mussel feeder.

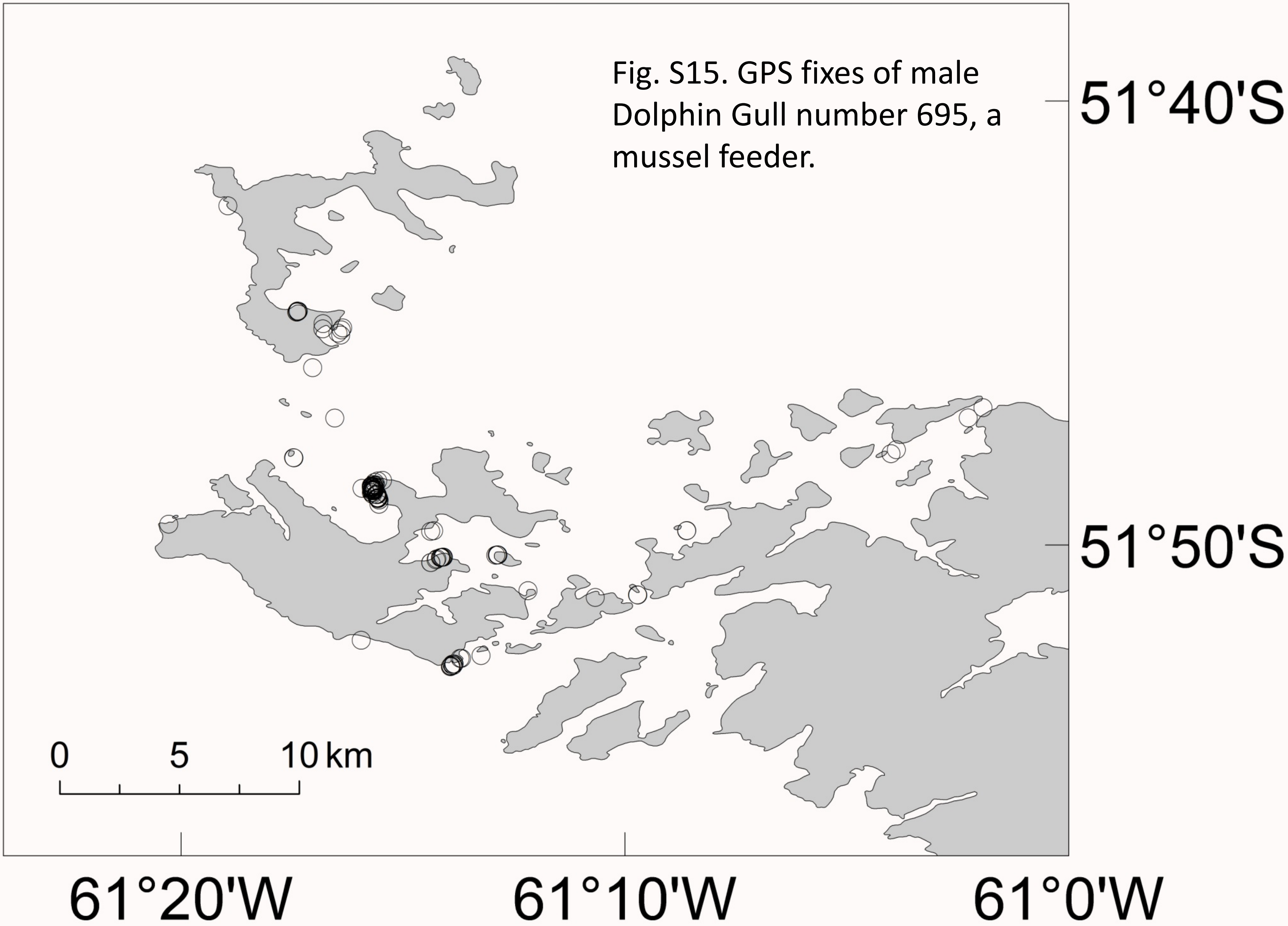

Fig. S15. GPS fixes of male Dolphin Gull number 695, a mussel feeder.

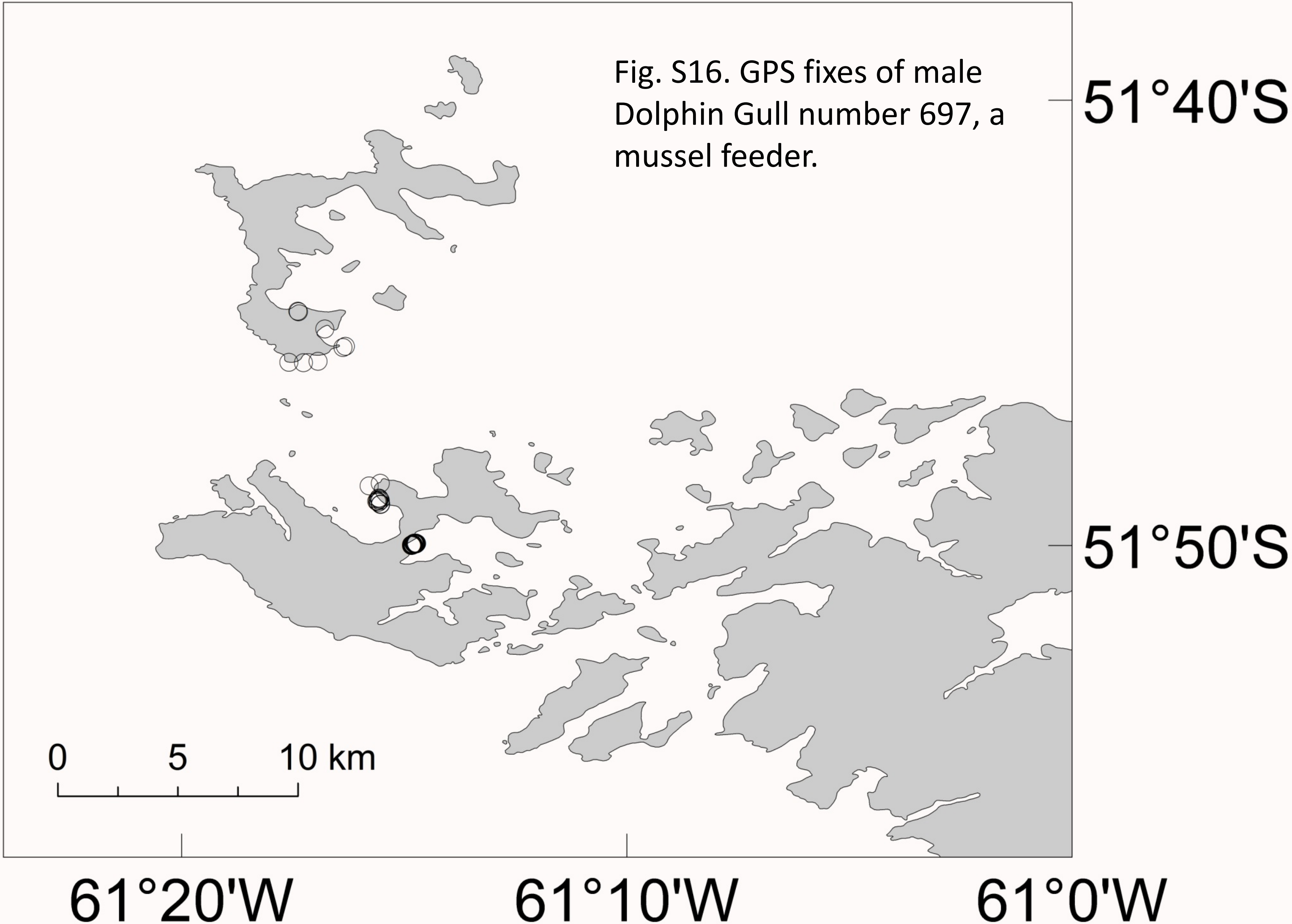

Fig. S16. GPS fixes of male Dolphin Gull number 697, a mussel feeder.

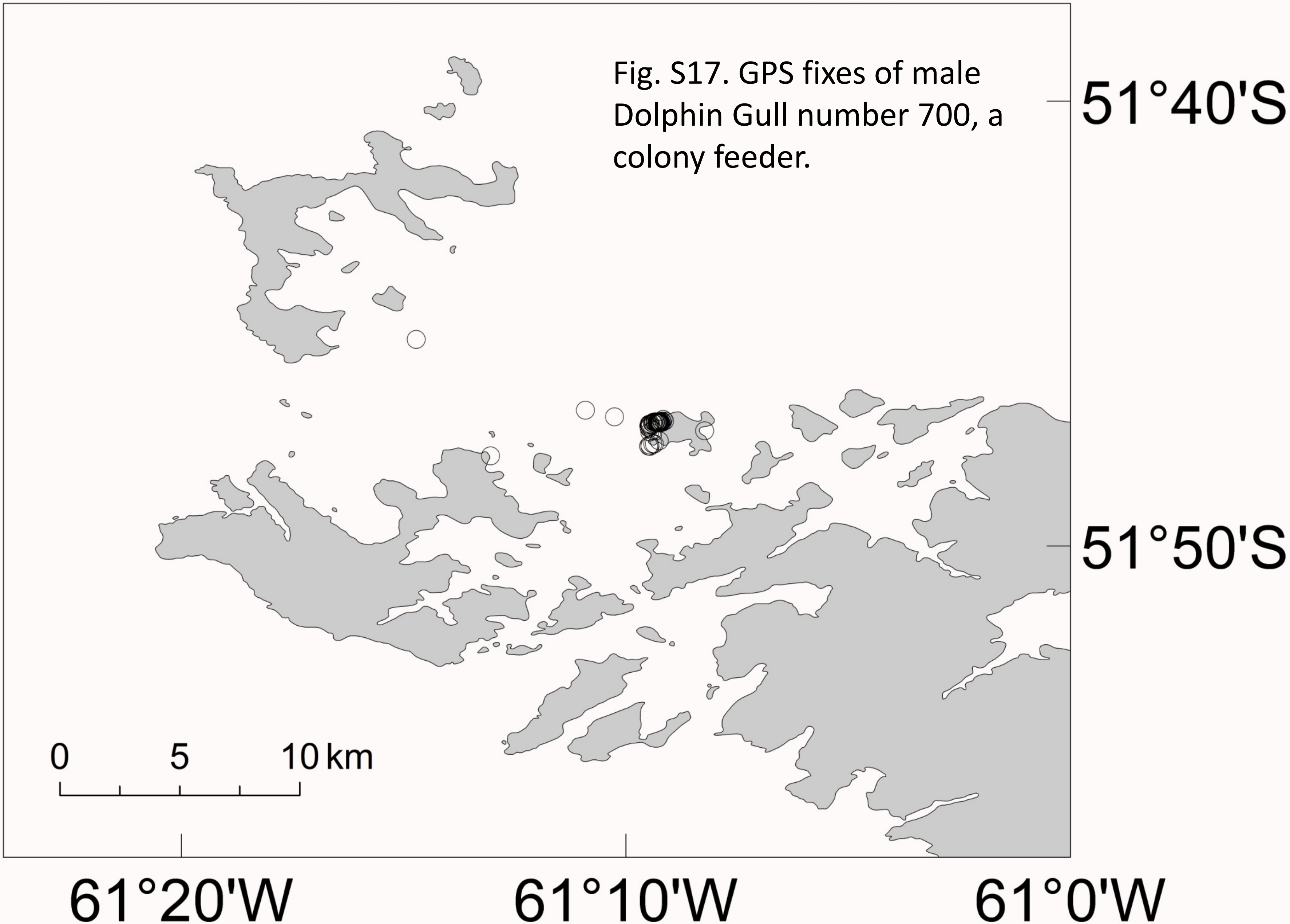

Fig. S17. GPS fixes of male Dolphin Gull number 700, a colony feeder.
