# Supplementary material for: Distribution Patterns Predict Individual Specialization in the Diet of Dolphin Gulls
Source: PLoS One. 2013 Jul 2;8(7):e67714. doi: 10.1371/journal.pone.0067714 (PMC3699636; doi:10.1371/journal.pone.0067714)
Supplement: File S3 — Figure S18–Figure S19. Figure S18. Kernel density analyses of GPS data of Dolphin Gulls Leucophaeus scoresbii. The 50, 60, 70, 80, 90 and 95% density contour areas of tagged dolphin gulls that repeatedly attended seabird and seal colonies (denoted as ‘colony feeders’). GPS locations of colony feeders are marked with triangles. Mussels, seals and seabirds present per site: site 1) Imperial Shags Leucocarbo atriceps, Rockhopper Penguins Eudyptes chrysocome, Black-browed Albatrosses Thalassarche melanophris; site 2) Imperial Shags and Rock Shags Phalacrocorax magellanicus, Fur Seals Arctocephalus australis; site 3) Rock Shags; site 4) Blue Mussel Mytilus edulis chilensis; site 5) Imperial and Rock shags; site 6) Imperial and Rock shags, Southern Giant-petrels Macronectes giganteus; site 7) Imperial and Rock shags. The Dolphin Gull colony at New I. is indicated with a white square. A second Dolphin Gull colony in the region is marked with a black square. Figure S19. Kernel density analyses of GPS data of Dolphin Gulls Leucophaeus scoresbii. The 50, 60, 70, 80, 90 and 95% density contour areas of tagged dolphin gulls that repeatedly attended mussel beds (denoted as ‘mussel feeders’). GPS locations of mussel feeders are marked with circles. Mussels, seals and seabirds present per site: site 1) Imperial Shags Leucocarbo atriceps, Rockhopper Penguins Eudyptes chrysocome, Black-browed Albatrosses Thalassarche melanophris; site 2 Imperial Shags and Rock Shags Phalacrocorax magellanicus, Fur Seals Arctocephalus australis; site 3) Rock Shags; site 4) Blue Mussel Mytilus edulis chilensis; site 5) Imperial and Rock shags; site 6) Imperial and Rock shags, Southern Giant-petrels Macronectes giganteus; site 7) Imperial and Rock shags. The Dolphin Gull colony at New I. is indicated with a white square. A second Dolphin Gull colony in the region is marked with a black square. (PDF) [file pone.0067714.s003.pdf]

Supporting information to:

Distribution patterns predict individual specialization in the diet of Dolphin Gulls

Juan F. Masello, Martin Wikelski, Christian C. Voigt, Petra Quillfeldt

Content:

Figure S18. Kernel density analyses of GPS data of Dolphin Gulls *Leucophaeus scoresbii.* The 50, 60, 70, 80, 90 and 95% density contour areas of tagged dolphin gulls that repeatedly attended seabird and seal colonies (denoted as 'colony feeders'). GPS locations of colony feeders are marked with triangles. Mussels, seals and seabirds present per site: site 1) Imperial Shags *Leucocarbo atriceps*, Rockhopper Penguins *Eudyptes chrysocome*, Black-browed Albatrosses *Thalassarche melanophris*; site 2) Imperial Shags and Rock Shags *Phalacrocorax magellanicus*, Fur Seals *Arctocephalus australis*; site 3) Rock Shags; site 4) Blue Mussel *Mytilus edulis chilensis*; site 5) Imperial and Rock shags; site 6) Imperial and Rock shags, Southern Giant-petrels *Macronectes giganteus*; site 7) Imperial and Rock shags. The Dolphin Gull colony at New I. is indicated with a white square. A second Dolphin Gull colony in the region is marked with a black square.

Figure S19. Kernel density analyses of GPS data of Dolphin Gulls *Leucophaeus scoresbii.* The 50, 60, 70, 80, 90 and 95% density contour areas of tagged dolphin gulls that repeatedly attended mussel beds (denoted as 'mussel feeders') . GPS locations of mussel feeders are marked with circles. Mussels, seals and seabirds present per site: site 1) Imperial Shags *Leucocarbo atriceps*, Rockhopper Penguins *Eudyptes chrysocome*, Black-browed Albatrosses *Thalassarche melanophris*; site 2 Imperial Shags and Rock Shags *Phalacrocorax magellanicus*, Fur Seals *Arctocephalus australis*; site 3) Rock Shags; site 4) Blue Mussel *Mytilus edulis chilensis*; site 5) Imperial and Rock shags; site 6) Imperial and Rock shags, Southern Giant-petrels *Macronectes giganteus*; site 7) Imperial and Rock shags. The Dolphin Gull colony at New I. is indicated with a white square. A second Dolphin Gull colony in the region is marked with a black square.

Fig. S18

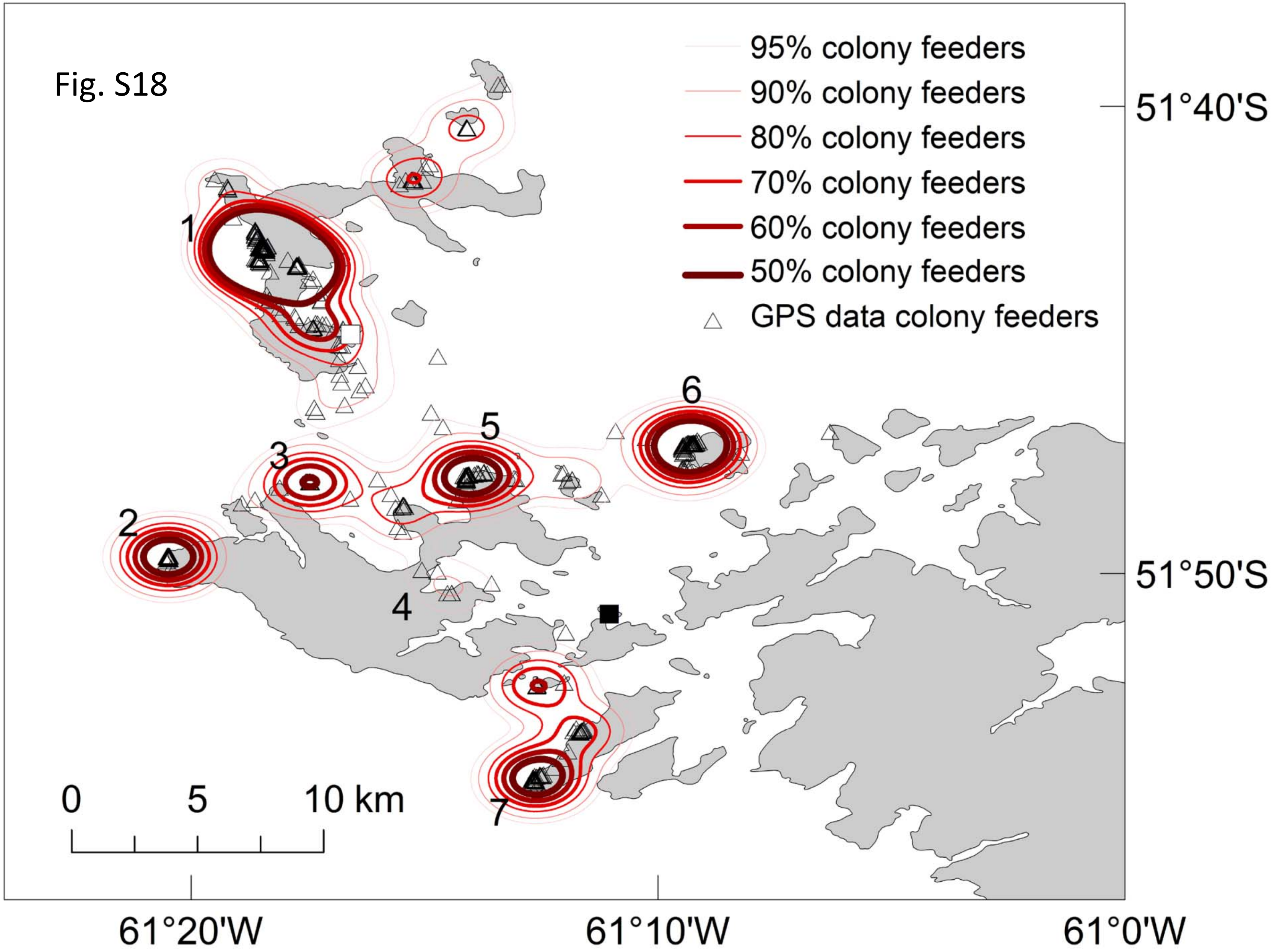

95% colony feeders
90% colony feeders
80% colony feeders
70% colony feeders
60% colony feeders
50% colony feeders
GPS data colony feeders
1
2
3
4
5
6
7
0
5
10 km
51°40'S
51°50'S
61°20'W
61°10'W
61°0'W

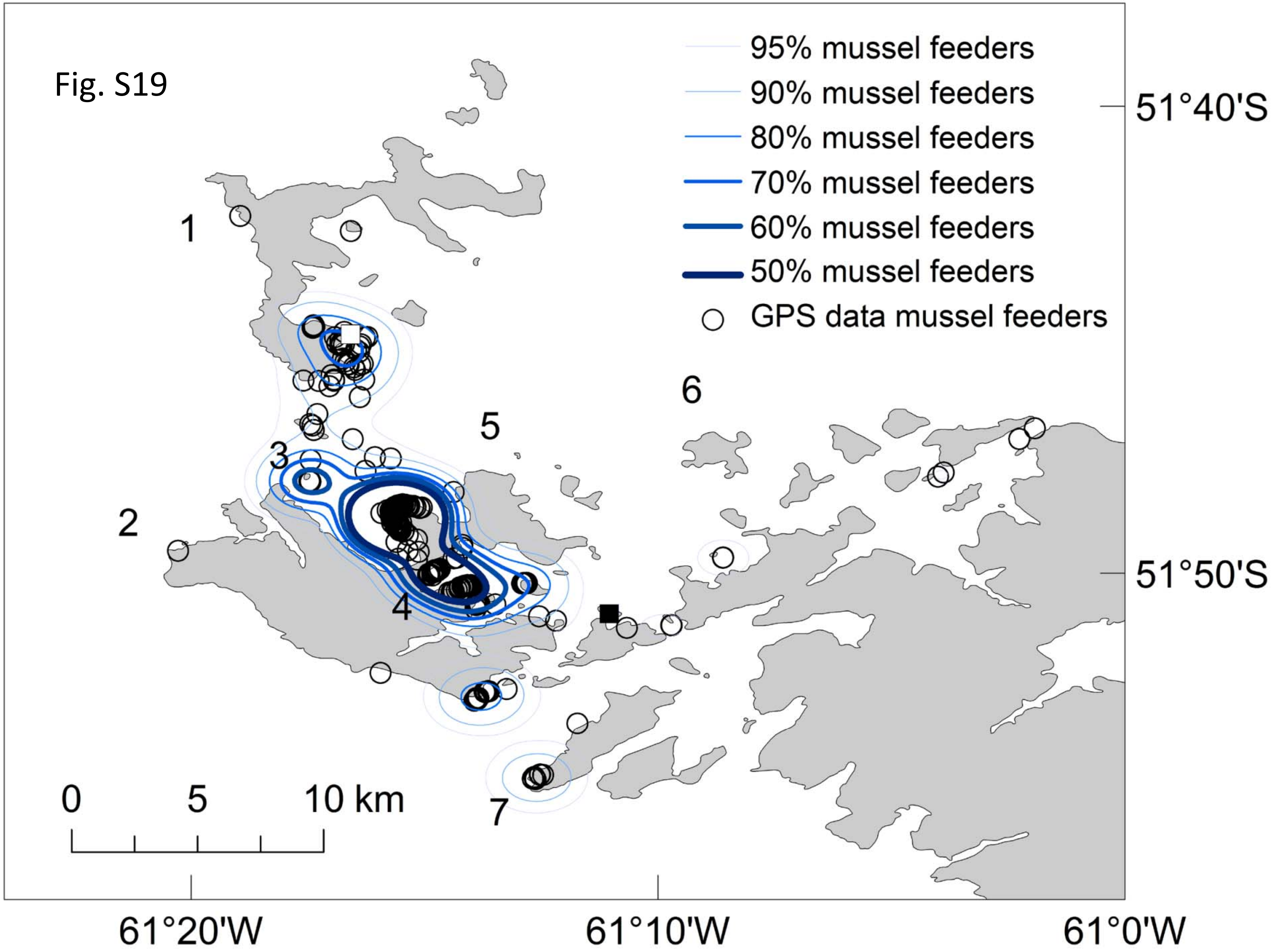
Fig. S19
95% mussel feeders
90% mussel feeders
80% mussel feeders
70% mussel feeders
60% mussel feeders
50% mussel feeders
GPS data mussel feeders
1
2
3
4
5
6
7
0
5
10 km
51°40'S
51°50'S
61°20'W
61°10'W
61°0'W
