# Supplementary material for: Distribution Patterns Predict Individual Specialization in the Diet of Dolphin Gulls
Source: PLoS One. 2013 Jul 2;8(7):e67714. doi: 10.1371/journal.pone.0067714 (PMC3699636; doi:10.1371/journal.pone.0067714)
Supplement: File S4 — Figure S20–S23. Figure S20. Feeding in seabird colonies. Most dolphin gulls Leucophaeus scoresbii (Traill, 1823) at New Island feed on the scraps of food dropped while larger seabirds such as imperial cormorants Leucocarbo atriceps (King, 1828) (upper picture) or gentoo penguins Pygoscelis papua (J. R. Forster, 1781) (lower picture) feed their chicks. Usually, dolphin gulls walk or fly close to a cormorant or penguin while feeding chicks. When the birds interrupt their feeding to attack the gull, they commonly drop some food, which is then picked up by the gull. Figure S21. Feeding in mussel beds. Dolphin gulls feed in mussel beds (upper left picture) by picking out blue mussels (Mytilus edulis chilensis), which they open by dropping them from some height unto the rocks below (lower picture). The mussel shells with holes are regularly found as a result (upper right picture). Figure S22. Feeding in mussel beds Dense beds or mats of Blue Mussel Mytilus edulis chilensis are common in the Falkland Islands/Islas Malvinas but largely confined to sheltered bays and inlets. A particularly large Blue Mussel bed is located in Beaver Harbour, on the east side of Beaver Island (pictures below; see also Fig. 1). Figure S23. Additional food source. Occasionally, dolphin gulls picked up krill larvae or other invertebrates that washed ashore after storms in the intertidal zone. (PDF) [file pone.0067714.s004.pdf]

Supporting information to:

Distribution patterns predict individual specialization in the diet of Dolphin Gulls

Juan F. Masello, Martin Wikelski, Christian C. Voigt, & Petra Quillfeldt

Figure S20. Feeding in seabird colonies: Most dolphin gulls *Leucophaeus scoresbii* (Traill, 1823) at New Island feed on the scraps of food dropped while larger seabirds such as imperial cormorants *Leucocarbo atriceps* (King, 1828) (upper picture) or gentoo penguins *Pygoscelis papua* (J. R. Forster, 1781) (lower picture) feed their chicks. Usually, dolphin gulls walk or fly close to a cormorant or penguin while feeding chicks. When the birds interrupt their feeding to attack the gull, they commonly drop some food, which is then picked up by the gull.

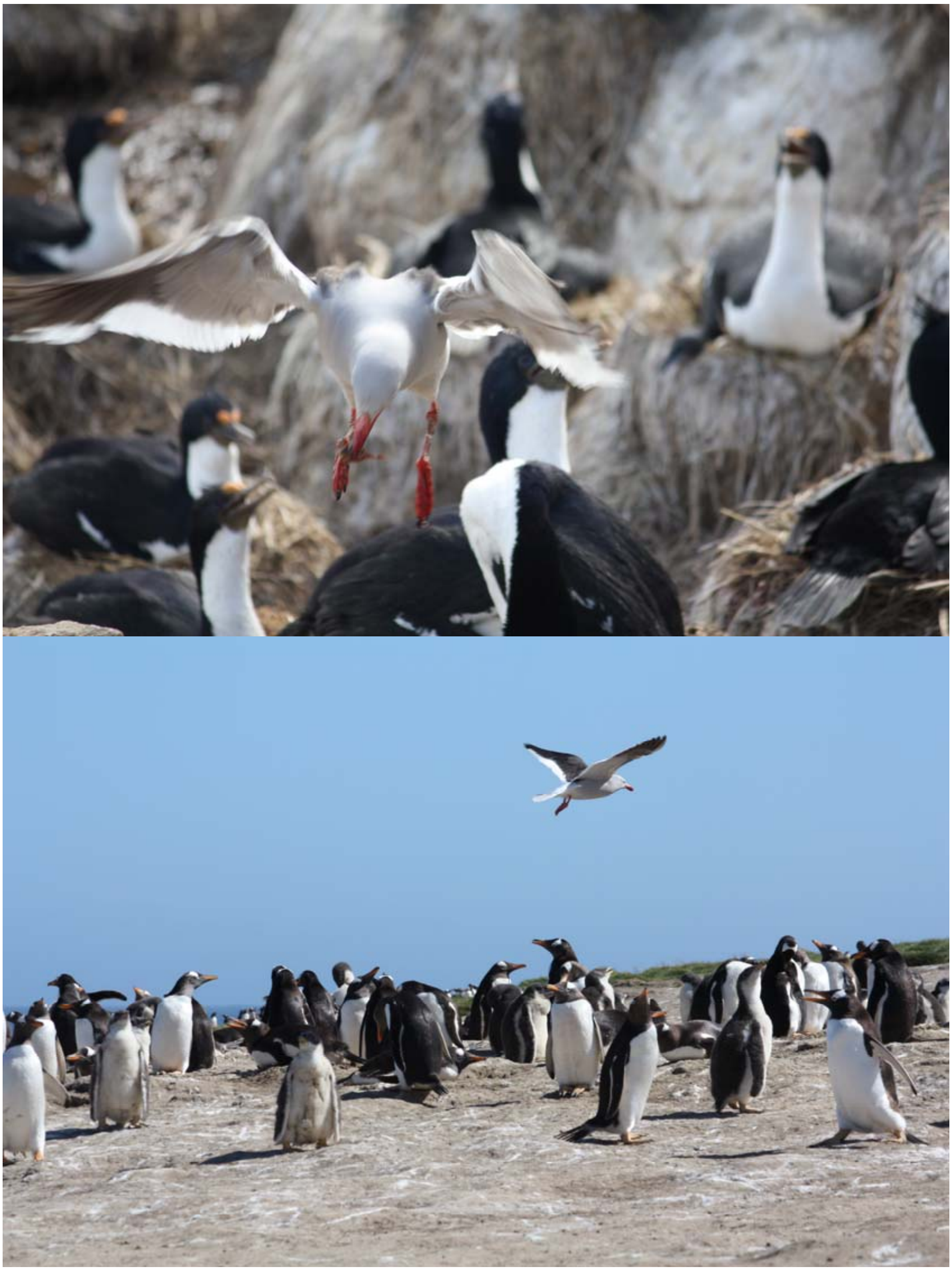

Figure S21. Feeding in mussel beds: Dolphin gulls feed in mussel beds (upper left picture) by picking out blue mussels (*Mytilus edulis chilensis*), which they open by dropping them from some height unto the rocks below (lower picture). The mussel shells with holes are regularly found as a result (upper right picture).

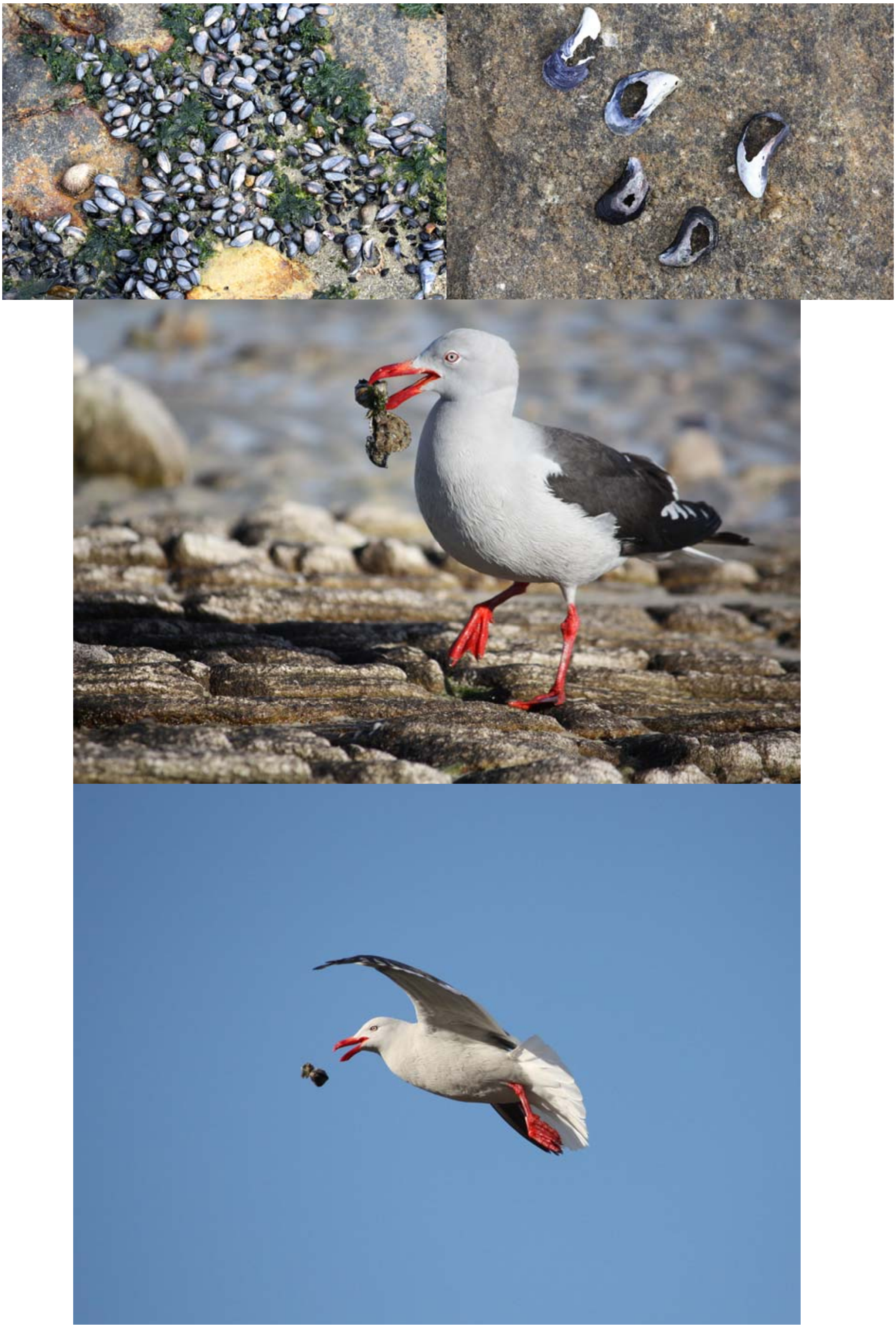

Figure S22. Dense beds or mats of Blue Mussel *Mytilus edulis chilensis* are common in the Falkland Islands / Islas Malvinas but largely confined to sheltered bays and inlets. A particularly large Blue Mussel bed is located in Beaver Harbour, on the east side of Beaver Island (pictures below; see also Fig. 1).

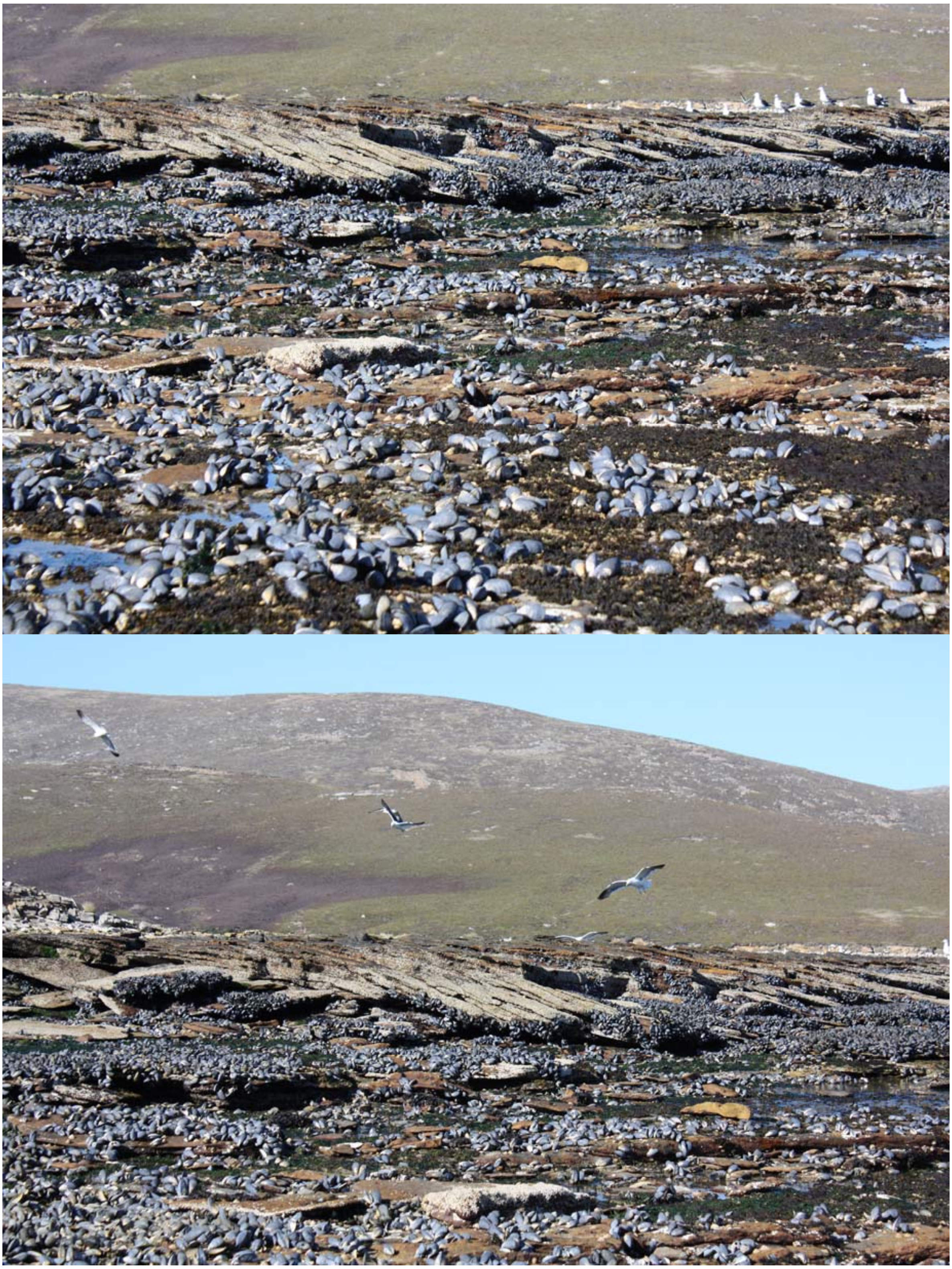

Figure S23. Additional food source: Occasionally, dolphin gulls picked up krill larvae or other invertebrates that washed ashore after storms in the intertidal zone.

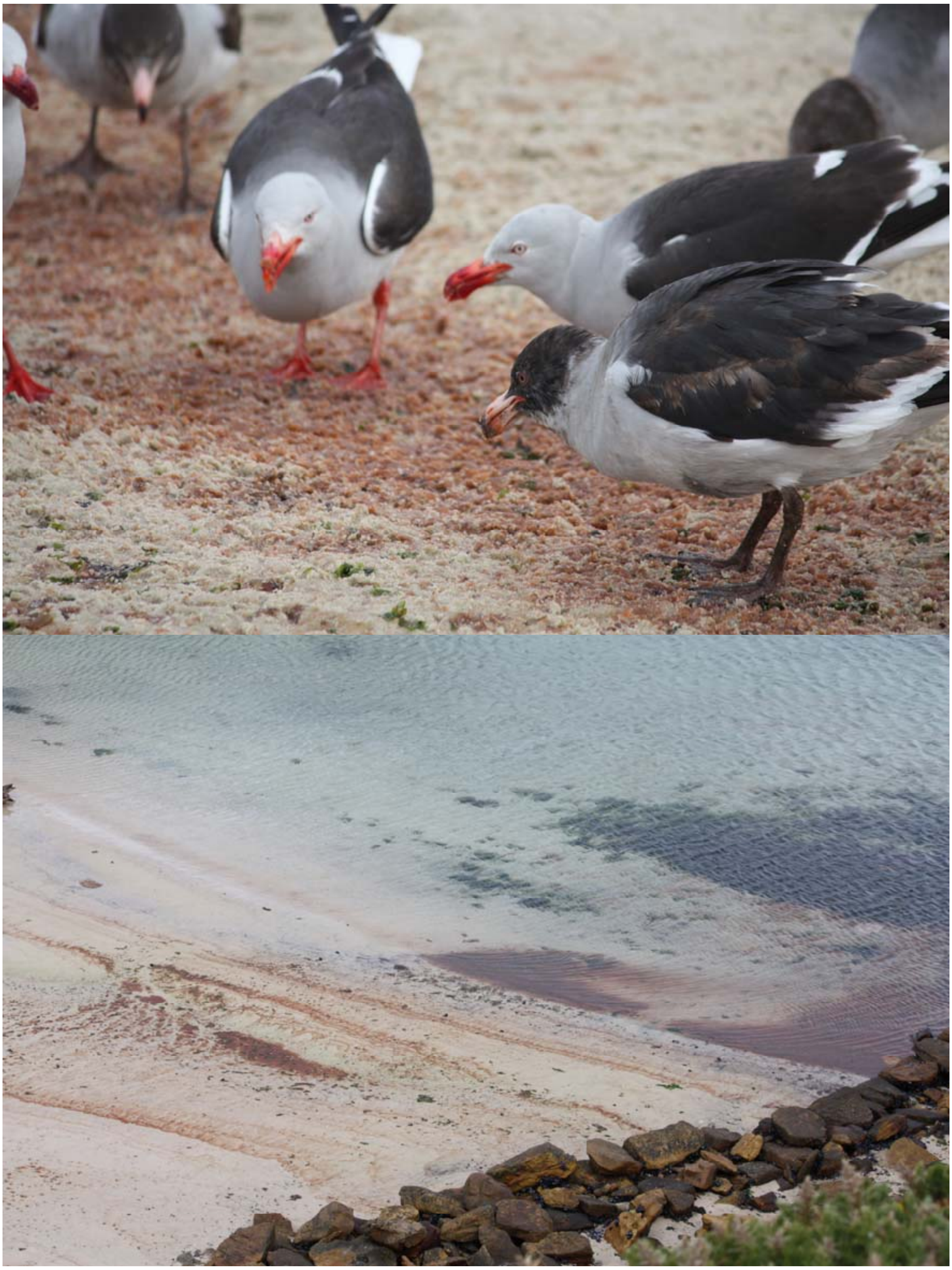

All pictures by Petra Quillfeldt
